# Targeting the ERK Signaling Pathway to Halt Adventitia Remodeling of Ascending Thoracic Aortic Aneurysm

**DOI:** 10.1016/j.jacbts.2026.101665

**Published:** 2026-09-25

**Authors:** Khaled AK. Mohammed, Elisa Avolio, Valeria V. Alvino, Eltayeb Mohamed Ahmed, Cha Rajakaruna, Ahmed Ghoneim, Ahmed Elminshawy, Gianni D. Angelini, Paolo Madeddu

**Affiliations:** a Bristol Heart Institute, Translational Health Sciences, Bristol Medical School, University of Bristol, Bristol, United Kingdom; b Department of Cardiothoracic Surgery, Faculty of Medicine, Assiut University, Assiut, Egypt; c Bristol Heart Institute, Bristol Royal Infirmary, University Hospitals Bristol and Weston NHS Foundation Trust, Bristol, United Kingdom

**Keywords:** aorta adventitia, aorta aneurysm mouse model, aorta pericytes, ascending thoracic aorta aneurysm, MEK inhibitor, PD0325901

## Abstract

Ascending thoracic aortic aneurysms (AsTAAs) are life-threatening and lack effective drug treatments. This study investigated whether abnormal mitogen-activated protein kinase kinase/extracellular signal-regulated kinase (MEK/ERK) activation in adventitial pericytes contributes to aneurysm progression. Human AsTAA tissue showed vasa vasorum remodeling and displaced pericytes. Isolated AsTAA-pericytes showed increased MEK/ERK signaling, proliferation, migration, proteolytic activity, proinflammatory secretome, and altered angiogenic function. These changes were reversed by the MEK inhibitor PD0325901. In a mouse model, short-term MEKi treatment reduced aortic dilatation, improved compliance, preserved elastin, and lessened inflammation, without adverse effects. This is the first study identifying dysregulated adventitial pericytes as a hallmark of AsTAA, supporting MEKi as a promising therapeutic.

## Highlights

• Ascending thoracic aortic aneurysm (AsTAA) is life‑threatening and lacks drug therapy.
• AsTAA patients’ aortic adventitia shows vascular remodelling with displaced pericytes.
• AsTAA pericytes are dysfunctional. A MEK inhibitor (MEKi) rescued ERK1/2 activation.
• In a mouse AsTAA model, systemic MEKi reduces aortic dilation and improves compliance.
• MEKi may prevent AsTAA by targeting aneurysmal mechanisms and pericyte disruption.

## Visual Abstract

**Figure:**
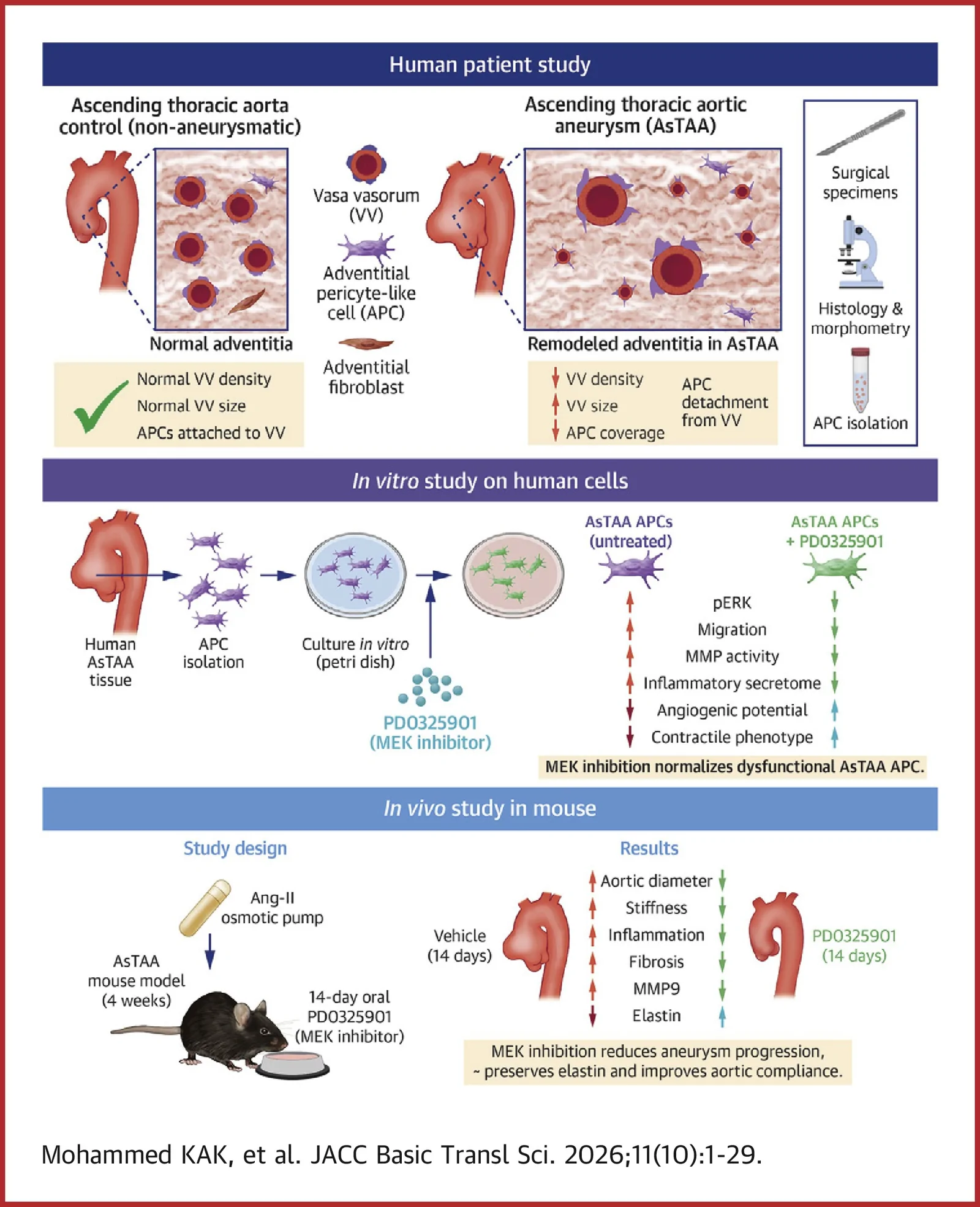

Aortic aneurysm (AA) is caused by the progressive weakening of the aorta wall and is frequently asymptomatic but can cause substantial mortality because of dissection and rupture.^1^^,^^2^ These complications result in almost 10,000-16,000 fatalities annually in the U.S. alone.^3^^,^^4^ Thoracic AAs (TAAs) and abdominal AAs (AAAs) exhibit distinct pathogenic characteristics and risk factors.^5^ Within TAAs, syndromic and more frequent nonsyndromic forms are recognized, depending on the presence or absence of genetic mutations and extra-aortic pathology.^6^ Sporadic nonfamilial forms constitute the majority of nonsyndromic TAAs.^7^^,^^8^ Smoking, cholesterol levels greater than 6.9 mmol/L, hypertension, and coronary artery disease were strong predictors of AAA diagnosis in multivariable analyses, whereas hypertension was the only significant predictor for TAAs.^5^ Interestingly, none of the above variables could forecast the occurrence of ascending TAAs (AsTAAs) in subgroup analysis.^5^ This variability in how risk factors predict outcomes points to distinct underlying causes of aneurysm development. Accordingly, previous research has shown that AsTAAs are neither morphologically nor microscopically atherosclerotic and, paradoxically, are associated with reduced low-density lipoprotein levels, a marker of atherosclerosis, after controlling for covariates.^9^, ^10^, ^11^ Differences in the embryologic origins of both medial and adventitial cells between the ascending and descending aorta further support this distinction, with cells in the ascending and arch aorta derived from the neural crest,^12^ the descending aorta arising from the paraxial mesoderm,^12^ whereas the abdominal part arises from the splanchnic mesoderm.^13^ Moreover, there is still no clear consensus on how to monitor and treat TAAs, in contrast to the well-established approaches for AAAs.^1^^,^^14^

Mechanistic research on TAAs has traditionally focused on degeneration of the medial layer as the primary therapeutic target. More recently, however, adventitial vasa vasorum (VV) have emerged as key contributors to the initiation and progression of aneurysm pathology.^15^^,^^16^ A plentiful VV network supplies the proximal aorta with enough perfusion to meet its substantial metabolic demands.^17^ VV loss and consequent hypoxia have a deleterious impact on aortic function, as demonstrated in animal models.^18^ Parietal stress because of chronic arterial hypertension could contribute to an adverse remodeling of VV, which becomes fragile, leaky, and prone to inflammation.^19^ Despite this initial evidence, the involvement of vasculogenic cells surrounding the VV has been overlooked. In previous studies, we showed that clonogenic cells carrying the classical neural glial cell antigen 2 (NG2)/platelet-derived growth factor beta (PDGFRβ) combination along with hematopoietic (CD34) and mesenchymal markers (CD44, CD90, CD105, CD29, CD49a, CD49b, CD13, CD59, and CD73), but negative for the endothelial antigen CD31, form a vasculogenic niche around the VV of the human adult and fetal aorta^20^^,^^21^ and vena saphena.^22^ These so-called adventitial pericyte-like cells (APCs) maintain vascular wall stability but, when dysfunctional, also contribute to adverse vascular remodeling.^22^^,^^23^ At the organismal level, pericyte loss is associated with vascular fragility, altered barrier function, and fibrotic remodeling,^24^^,^^25^ all of which are hallmarks of TAA. Despite their recognized importance in vascular biology, the mechanistic role of APCs in TAA pathogenesis remains poorly understood.

Overactivation of the mitogen-activated protein kinase (MAPK)/extracellular signal-regulated kinase (ERK) pathway is a key feature in TAAs.^26^^,^^27^ This phospho-relay signaling cascade, which regulates cellular proliferation, migration, and differentiation, has been consistently implicated in TAA pathophysiology.^28^, ^29^, ^30^, ^31^ The MAPK/ERK pathway consists of a 3-tiered kinase cascade, which transmits signals from the cell membrane to the nucleus. Once activated by phosphorylation through upstream mitogen-activated protein kinase kinases (MEK1/2), ERK1/2 translocates to the nucleus, where it phosphorylates multiple transcription factors (TFs) and drives changes in gene expression.^31^ Angiotensin II (Ang-II), a key mediator in TAA pathogenesis, stimulates ERK1/2 signaling through the angiotensin II type 1 receptor (AT1R). Activation of this pathway promotes vascular smooth muscle cell (VSMC) proliferation, matrix remodeling, and phenotypic differentiation of adventitial fibroblasts into myofibroblasts, ultimately favoring AA progression.^32^, ^33^, ^34^ Dysregulated ERK signaling in pericytes has been associated with impaired angiogenic function. In a previous study, pharmacologic inhibition of ERK reprogrammed human cardiac pericytes toward a proangiogenic phenotype, promoting recovery after myocardial infarction in mice.^35^ Here, we propose that a similar approach could protect the adventitial vasculogenic niche from adverse remodeling, thereby preserving the structure of the outer layers of the aorta and slowing down AsTAA progression.

Despite advances in imaging and surgical techniques, the absence of effective pharmacologic therapies to slow TAA progression underscores a major unmet clinical need.^36^ Surgical correction is recommended when the aorta reaches a size (5-5.5 cm) where the risk of complications (ie, acute dissection or fatal rupture) equals or exceeds the operative risk.^1^^,^^37^ However, this criterion depends on the underlying pathology, growth rate, family history, and individual patient.^1^ Many patients remain in the gray zone, where the aortic diameter remains below the hinge point for surgical intervention.

In this study, based on human aneurysm tissues, in vitro functional and molecular studies on APCs, and a nonatherosclerotic animal model of AsTAA, we show for the first time that malfunctioning VV-APCs are a typical AsTAA signature. We also introduce a targeted pharmacologic approach, PD0325901—a selective MEK inhibitor (MEKi)—to safely reprogram the human APC transcriptome, restore cell function, and halt aneurysm progression in mice.

## Materials and Methods

The number of biological replicates included in each experiment is reported in the figure legends.

### Ethical approval

This study complies with the guidelines of the Declaration of Helsinki. Human aortic samples were collected from the Bristol Heart Institute, Bristol, UK, in compliance with the waste tissue ethical protocol (REC reference: 06/Q2001/197).

The in vivo study was covered by a license from the British Home Office (PP1377882) and complied with the EU Directive 2010/63/EU. Experiments were carefully designed to meet the NC3Rs guidelines. Procedures were carried out according to the principles in the NIH Guide for the Care and Use of Laboratory Animals (National Academies Press, 2011). Termination was conducted according to humane methods outlined in the Guidance on the Operation of the Animals (Scientific Procedures) Act 1986 Home Office (2014). The report of results is in line with the Animal Research: Reporting of In Vivo Experiments (ARRIVE) guidelines.

### Sex as a biological variable

Studies on human aortic adventitia specimens and derived cells included both male and female donors. However, because of the small sample size, sex was not considered as a biological variable.

In animal studies, only male mice were used, as the modeled disease is more prevalent and severe in males, making them a more relevant choice for studying TAA progression and evaluating potential treatments.^29^^,^^38^ This approach enhanced experimental consistency and reproducibility, while also reducing the required sample size in accordance with the NC3Rs guidelines.

### Human aortic samples collection

Full aortic layers (ie, intima, media, and adventitia) were collected from patients undergoing surgical correction of AsTAA. *Inclusion criteria*: 1) Sporadic AsTAA, 2) diameter >5 cm, and 3) intact aortic valve. *Exclusion criteria*: 1) patient's unacceptance to participate, 2) bicuspid aortic valve, 3) acute/chronic aortic dissection, either spontaneous or related to trauma, aortitis, or infection, and 4) genetic syndromes (eg, Marfan syndrome). Specimens of the adventitia layer were obtained from patients with normal average ascending aorta diameter undergoing coronary artery bypass graft surgery. Supplemental Tables 1 and 2 summarize salient patient data and the specific use of aortic samples in experiments. Each specimen, whenever sufficient, was divided into 2 parts, 1 used for histologic studies and the other for APC isolation and expansion.

### Histology and immunohistochemistry on human samples

Freshly collected samples were immediately fixed by incubation in 4% w/v paraformaldehyde (PFA, Sigma-Aldrich) at 4 °C for 24 hours. Following fixation, the samples were processed for histology, embedded in paraffin, and sectioned into 5-μm-thick slices, which were mounted onto microscopy slides (Thermo Fisher). Slices were then dewaxed, rehydrated, and subjected to heat-retrieval using citrate buffer (0.01 M, pH 6, for 30 minutes at 98 °C – Sigma-Aldrich). For morphometric analysis, sections were stained with hematoxylin and eosin and Van Gieson stain. Immunohistochemistry (IHC) sections were incubated with 5% v/v donkey serum to prevent nonspecific antibody binding and minimize the background signal. They were then incubated overnight at 4 °C with primary antibodies (Supplemental Table 3). Incubation with donkey Alexa-Fluor 488, 568, or 647-conjugated secondary antibodies (all from ThermoFisher) was carried out at room temperature for 1 hour, followed by nuclear staining with 4′,6-diamidino-2-phenylindole and mounting with Fluoromount-G (Thermo Fisher Scientific, # 00-4958-02). Imaging was done using an Axio Observer Z1 fluorescent microscope (Zeiss). Quantification included counting the number of VV per imaging field and assessing the VV wall thickness, lumen diameter, and area, according to the published method of Billaud et al.^15^ The entire adventitial area was analyzed for each sample.

### APC isolation, culture, and expansion

APCs were isolated using an explant outgrowth method.^39^ Briefly, aortic adventitial samples were cut into small fragments (approximately 3 mm^2^) and seeded onto tissue culture flasks or 6-well plates. These fragments were cultured in full Endothelial Cell Growth Media (ECGM2, PromoCell) supplemented with 5% fetal bovine serum (FBS) and maintained in an incubator set at 37 °C with 5% CO_2_. We previously demonstrated that ECGM2 is the optimal medium to achieve selective APC expansion from human vascular tissue, excluding the growth of contaminant cells and maintaining cell viability.^40^ The medium was replenished every 48 hour, and cellular outgrowth was observed sprouting from the adventitial explants after 5-7 days of incubation, showing the characteristic spindle-shape of pericytes (Supplemental Figures 1A and 1B). Because >95% of cells were positive for pericyte markers PDGFRβ and NG2, further immunosorting to separate pericytes from other adventitial cells was not required. Once the cells reached confluence, the tissue explants were removed, and cells were detached using Acutase solution (Sigma, A6964) for subculturing and expansion. All experiments were conducted using cells between passages 3 and 6.

### APC doubling time, viability, and proliferation

A 6-well tissue culture plate was used to seed 30,000 cells per well, which were then maintained in full ECGM2 media within an incubator set at 37 °C and 5% CO_2_. Cells were detached and counted on days 4, 5, 6, and 7 to generate a growth curve. Cell counting was performed using trypan blue (Lonza, # 17-942E) and a Bürker counting chamber (Hawkslev). The number of viable cells was recorded to calculate the cell doubling time. Cell viability was assessed with Calcein-AM/Ethidium homodimer (EthDIII) staining (Biotium, Cat# 30002) according to the manufacturer's instructions. Proliferation was assessed using the Click-iT EdU Cell Proliferation Kit (C10337 - ThermoFisher Scientific) according to the manufacturer's instructions and an anti-Ki67 antibody (Supplemental Table 4). All experiments were performed in duplicate.

### Immunocytochemistry

A 96-well black plate with a clear bottom was used to seed 5,000 cells per well, followed by incubation in full ECGM2 media at 37 °C in a 5% CO_2_ incubator. After 48 hours, cells were fixed with 4% v/v PFA, permeabilized, and incubated with 5% FBS for 1 hour to minimize nonspecific antibody binding. Subsequently, cells were incubated overnight at 4 °C with primary antibodies (Supplemental Table 4). After washing with PBS, cells were incubated with donkey Alexa-Fluor 488, 568, or 647-conjugated secondary antibodies (all from Thermo Fisher Scientific) at room temperature in the dark for 1 hour. Nuclear counterstaining was performed using 4′,6-diamidino-2-phenylindole, and cells were preserved using Fluoromount-G (Thermo Fisher Scientific, # 00-4958-02). Imaging was conducted using an inverted Axio Observer Z1 Fluorescence Microscope (Zeiss). All experiments were performed in technical duplicates.

### Analysis of the APC secretome by enzyme-linked immunosorbent assay

After cultured cells reached 80%-90% confluence, the medium was removed, and cells were rinsed with Dulbecco's Phosphate Buffered Saline. Cells were then incubated with basal ECBM2 media (ie, serum-free and growth factors-free media) for 48 hours. The conditioned medium was collected, centrifuged to remove floating cells and debris, and stored at −80 °C until analysis. Cells were detached and counted to normalize the levels of secreted factors against the number of cells. Enzyme-linked immunosorbent assay (ELISA kits (R&D Systems) were used to quantify secreted angiogenic factors, including angiopoietin-1 (ANGPT-1, #DY923) and ANGPT-2 (#DY623) and thrombospondin-1 (TSP-1, #DY3074), extracellular matrix (ECM) proteins Fibronectin (#DY1918) and Collagen 1-alpha (#DY6220), and proinflammatory factors interleukin-6 (IL-6, #DY206) and macrophage chemoattractant protein 1 (MCP-1, #DY279). The levels of secreted factors were normalized against the final total cell number and time. Absorbance was measured using a GloMax Discover System. All samples were assayed in technical duplicates.

### Angiogenic tube formation assay

The ability of isolated APCs to interact with and support the angiogenic tube networks formed by aortic endothelial cells (AoECs) was assessed. APCs were cocultured with AoECs at a 2:5 ratio in ECBM2 media supplemented with 2% FBS. Cells were plated on a thin layer of ECM gel (Sigma # 6909) within the wells of angiogenesis slides (Ibidi, # 81506). They were then maintained for 6 hours at 37 °C in a 5% CO_2_ incubator before being imaged using a brightfield microscope (Leica). Experiments were performed in triplicate, and the total tube length per image was measured using ImageJ Software. In initial experiments, to verify the direct physical interaction between APCs and AoECs, APCs were labeled with the red fluorescent tracker Vybrant DiI Cell-Labeling Solution (Invitrogen) according to the manufacturer's instructions.

### Wound closure migration assay

A wound closure migration assay was conducted to evaluate the migratory capacity of isolated APCs. In this assay, 15,000 cells were seeded into each well of a 96-well plate. Once the cells reached confluence, a scratch was created in the center of the well using a 20 μL pipette tip. Cells were then incubated in basal (ie, serum- and growth factor-free) ECBM2 media supplemented with hydroxyurea (2 mM) to inhibit proliferation. Human recombinant platelet-derived growth factor BB (PDGF-BB, Peprotech, 20 ng/mL) was used to stimulate cell migration. Absence of growth factors was used as a control. Images of the scratch area were taken at 0, 6, 8, and 10 hours using an inverted microscope (Leica). The scratch/wound area was quantified using ImageJ software. For each biological sample, 4 technical replicates were included.

### In vitro invasion assay

To test the APCs' ability to migrate through a semipermeable membrane coated with a thin ECM layer in response to the chemoattractant PDGF-BB, we used the QCM 96-Well Cell Invasion Assay (ECM 555, Millipore). The kit includes a Boyden chamber coated with collagen (mimicking the basement membrane). A total of 40,000 APCs were seeded onto each membrane and resuspended in serum-free and growth factor-free ECBM2 media. PDGF-BB was added to the lower chamber to induce cell migration, using the same medium as in the upper chamber. Absence of PDGF-BB served as a control. After 24 hours of incubation, nonmigrated APCs on the top of the membranes were removed. Membranes and invaded cells were fixed with 4% (w/v) PFA and stained with a solution of 0.5% (w/v) Crystal Violet in 20% methanol (filtered through a 0.45 μm strainer) for 30 minutes at room temperature (20-25 °C). The wells were rinsed thoroughly with distilled water until all unbound stains were removed, and the plate was allowed to air-dry overnight. Finally, the dye was solubilized with 10% acetic acid, and the absorbance was measured at 600 nm using a spectrophotometer. The assay was performed in 5 technical replicates for each biological sample.

### Gelatinase assay

The gelatinase activity of the isolated APCs, specifically MMP-2 and MMP-9, was evaluated using a commercial kit (Sigma-Aldrich, # MAK348) according to the manufacturer's protocol. In this assay, 100,000 cells were seeded per well in a 6-well culture plate. Once confluent, cells were lysed using the provided cell lysis buffer. The lysate was then centrifuged at 16,000 x *g* for 10 minutes at 4 °C, and the supernatant was collected into an Eppendorf tube on ice. Protein concentration in the supernatant was determined using the Bicinchoninic acid Assay kit (Thermo Scientific, # 23250). Equal protein concentrations (20 μg) from each sample were loaded into the wells of a 96-well black plate. Reconstituted gelatin substrate was added to each well, and fluorescence was measured using a fluorescence microplate reader (GloMax). Gelatinase activity was expressed in units per milligram (U/mg), where 1 U is defined as the amount of gelatinase required to cleave the gelatin substrate per min. Experiments were performed in duplicate.

### Quantitative reverse transcription polymerase chain reaction

Upon confluence, cells were lysed using Qiazol (Qiagen, # 1023537), and total RNA was extracted using the RNeasy Plus kit (Qiagen, # 74134). After quantification, equal levels of RNA were reverse transcribed into cDNA using an RNA-to-cDNA kit (Applied Biosystems, # 4387406). Quantitative reverse transcription polymerase chain reaction (RT-qPCR) was then used to quantify the mRNA expression using TaqMan Master Mix and a Quant-Studio 5 System (Applied Biosystems). The relative gene expression was calculated using the 2^−ΔΔCt^ mathematical method.^41^ The list of TaqMan probes used for qPCR analysis is provided in (Supplemental Table 5). The qPCR was performed in triplicate.

### Western blotting

Confluent cells were rinsed with Dulbecco's Phosphate Buffered Saline and lysed with radioimmunoprecipitation assay buffer (Sigma, #R0278) supplemented with phosphatase and protease inhibitors (Sigma, #P5726 and P8340). Protein concentration was quantified using the Bicinchoninic acid Protein Assay kit (Thermo Scientific, # 23250). Equal amounts of total protein (10-20 μg) from each sample were loaded onto TGX 7.5% gels (Bio-Rad, # 4561024), and separated via electrophoresis using running buffer (Invitrogen, # LC2675) for 90 minutes at 110-150 V (PowerPac HC, Bio-Rad). Proteins were transferred to polyvinylidene fluoride (PVDF) membranes (Bio-Rad, #1620177) using transfer buffer (Novex, # LC3675) at a constant current of 0.25 A for 60 minutes. Membranes were washed and blocked for 2 hours in a solution of 5% w/v globulin-free bovine serum albumin (Sigma, # A3059) in Tris-buffered saline solution (Bio-Rad, # 1706435) + 0.05% v/v Tween-20 (Sigma, #P1379). Membranes were then incubated overnight at 4 °C with primary antibodies, including ERK 1/2, P-ERK 1/2 and β-Tubulin (Supplemental Table 6). The following day, membranes were washed and incubated at room temperature for 1 h with horseradish peroxidase (HRP)-conjugated anti-rabbit (GE Healthcare, # NA934 V) secondary antibody. After washing, protein bands were visualized using a Chemi-Doc MP system (Bio-Rad) following enhancement of chemiluminescence (ECL) signals with ECL detection Reagents (Cytiva, # PRN2232) for 5 minutes. Band densitometry was analyzed using ImageJ software. The densitometry values for the target proteins were normalized against β-Tubulin loading controls.

### PD0325901 treatment on cultured APCs

Confluent APCs were treated with the selective MEKi PD0325901 (250 nM, Sigma-Aldrich, #PZ0162) or vehicle (dimethyl sulfoxide; DMSO, same volume as MEKi, Sigma-Aldrich, # 20-139) for 10 d, with media being replaced every 48 hours. The MEKi concentration and treatment protocol were previously optimized by our group to ensure complete inhibition while preserving pericyte viability.^35^ At the end of the 10-day treatment period, cells were used for antigenic and functional characterization using the methods described previously.

### Next-generation RNA-sequencing

Strand-specific RNA sequencing was performed on total RNA by GENEWIZ (Azenta Life Sciences). In an initial study designed to assess the impact of disease on APCs, n = 3 AsTAA-APCs were compared with n = 3 control APCs. In a subsequent study, the effects of MEKi treatment on AsTAA-APCs were evaluated. Cells were conditioned for 10 days with PD0325901 (250 nM, as described above) or an equivalent volume of DMSO (vehicle control).

RNA sequencing was performed on an Illumina NovaSeq platform using a 2 × 150 bp configuration, generating approximately 20 million reads per sample. Raw reads were trimmed to remove adapter sequences and low-quality bases using Trimmomatic v0.36. Trimmed reads were aligned to the Homo sapiens GRCh38 reference genome (Ensembl) using STAR v2.5.2 b, and binary alignment/map files were generated. Gene-level counts were obtained using featureCounts from the Subread package v1.5.2, considering only uniquely mapped reads overlapping exon regions. The resulting count matrix was used for downstream differential expression analysis in DESeq2. Gene expression differences between groups were assessed using the Wald test to generate *P*-values and log2 fold changes. Genes with a false discovery rate < 0.05 and an absolute log2 fold change >1 were considered differentially expressed genes (DEGs). Gene ontology (GO) enrichment analysis was performed on significantly DEGs using GeneSCF v1.1-p2. The goa_human GO database was used to group genes by biological processes and assess statistical enrichment.

Extended gene annotations, hierarchical clustering, mean average plots, pathway analyses, heatmaps, and lollipop plots were generated using iDEP v2.4.4^42^ and Morpheus (https://software.broadinstitute.org/morpheus/). Protein–protein interaction networks were generated using STRING (https://string-db.org/).

The dataset has been deposited in NCBI's Gene Expression Omnibus and is accessible through the accession number GSE328194).

### *In vivo* treatment with MEKi in a mouse nonatherosclerotic TAA model

The in vivo effects of PD0325901 were evaluated in a mouse model of ANG-II-induced TAA as previously described.^43^^,^^44^ The experiment was designed using the NC3Rs Experimental Design Assistant. Using ascending aortic diameter as the primary endpoint, a mean difference of 0.25 mm was assumed, representing the biologically relevant reduction in diameter expected in the MEKi compared with the Vehicle group. Assuming a standard deviation (SD) of 0.14 mm, 80% power, and a 2-sided significance level of 0.05, 7 mice per TAA group were required. For the control vs TAA-Vehicle comparison, a mean difference of at least 0.3 mm was assumed, requiring only 5 mice in the control group.

In brief, basal echocardiography was performed before osmotic minipumps (Model 2004; ALZET) were implanted under recovery anesthesia using 2%-3% inhaled isoflurane in 14, 8-week-old male C57BL/6J mice (Charles River) for continuous subcutaneous ANG-II infusion (rate = 1,000 ng/kg/min, Enzo Life Sciences, #ALX-151-039). Seventy-two hours postimplantation, mice were randomly assigned to 2 groups: the treatment group received oral PD0325901 at a dose of 10 mg/kg per day (n = 7), and the vehicle group received oral DMSO (n = 7) for 14 d. The drug and control solvent (same volume as MEKi) were embedded in strawberry-flavored sugar-free jelly as previously described.^35^ Final echocardiography was conducted before humane euthanasia, followed by blood and organ collection. A control group (n = 5) of mice that did not receive ANG-II or DMSO/PD0325901 was included for reference control. Harvested aorta samples were fixed in 4% PFA at 4 °C overnight, immersed in 30% w/v sucrose in PBS overnight, and embedded in optimal cutting temperature compound for cryopreservation and histologic analyses. For western blotting, freshly frozen aorta samples were homogenized in radioimmunoprecipitation assay buffer supplemented with protease and phosphatase inhibitors (Sigma-Aldrich) using gentle MACS M tubes (Miltenyi). Tissue lysates were centrifuged at 10,000 *g*, at 4 °C, for 15 minutes, and processed as described above for detection of P-ERK/ERK protein levels.

### Histologic analysis of mouse aorta samples

Five-μm-thick frozen aorta sections were postfixed and permeabilized with ice-cold acetone (VWR) for 5 minutes at −20 °C and air-dried for 30 minutes. Sections were rehydrated with PBS for 10 minutes before proceeding with histologic and immuno-staining as described earlier for human samples. Antibody details are reported in Supplemental Table 3. Apoptosis was assessed using the in situ Direct DNA Fragmentation (TUNEL) Assay Kit (abcam ab66108). Collagen content was detected with the Azan Mallory staining as previously described.^45^ Active MMP9 was detected using Mouse and Rabbit Specific HRP_DAB IHC Detection Kit (abcam, ab236466). Because the MMP9 antibody was raised in mouse, we used the Mouse-on-Mouse Polymer IHC Kit (abcam, ab269452) to reduce the background staining caused by antimouse secondary antibody binding to endogenous mouse antibodies in mouse tissue. For each aorta sample, 2 entire slices were imaged and analyzed.

### Statistical analysis

Continuous data are presented as the mean ± SEM unless otherwise specified. The D'Agostino-Pearson and Kolmogorov-Smirnov normality tests were used to check if data were normally distributed. Groups were compared using Student's *t*-test or one-way analysis of variance (ANOVA) if normally distributed; otherwise, the Mann-Whitney *U* or Kruskal-Wallis test was used. Post hoc analyses included Tukey's and Dunn's multiple comparisons tests. Linear regression with comparison of slopes was used to evaluate whether the relationship between 2 continuous variables differed by group. For in vivo studies, baseline and final echocardiography parameters in the same animal were compared using paired tests. For all other comparisons between groups, unpaired tests were used. Statistical significance was defined as a *P* value <0.05, and analyses were conducted using GraphPad Prism version 11.0.0 (GraphPad Software).

## Results

### Adventitial remodeling is a signature of advanced human AsTAA

As illustrated in Supplemental Table 1, 5 of 9 AsTAA patients underwent graft replacement surgery, whereas the remaining also had a hemi-Arch graft replacement. All the controls underwent coronary artery bypass graft surgery for coronary artery disease. The 2 groups were similar in age and sex distribution. The aortic diameter was 5.8 ± 0.12 and 2.1 ± 0.13 cm in AsTAA and control, respectively (*P* < 0.001). Among the risk factors, arterial hypertension was highly prevalent in both groups, with no significant difference between the groups. Angiotensin-converting enzyme (ACE) inhibitors and angiotensin receptor antagonists (ARBs) were the main antihypertensive treatments in both groups. The number of patients under statin therapy was larger in the controls (66.7%) compared with the AsTAA group (22.2%), although the difference did not reach statistical significance (*P* = 0.14).

Analysis of hematoxylin and eosin and Van Gieson staining revealed remarkable adventitial vascular remodeling in AsTAA samples compared with controls, characterized by a larger size, wider lumens, and thicker walls (Figures 1A and 1B). The morphometric analysis of AsTAA samples demonstrated a significant reduction in VV density compared with controls (1.7 ± 0.3 vs 5.5 ± 0.6 per microscopic field, *P* = 0.005) (Figure 1C). Moreover, there was an 8-fold increase in the lumen area of AsTAA-VV compared to control-VV (1039 ± 163 vs 126 ± 25 μm^2^, *P* = 0.001) (Figure 1D). Also, AsTAA-VV are characterized by longer perimeters (Figure 1E). Normalizing the VV medial area to the lumen diameter resulted in AsTAA-VV having thicker walls (19.8 ± 1.5 μm^2^/μm) compared with controls (10.4 ± 0.9 μm^2^/μm, *P* = 0.001) (Figure 1F), which can be considered eccentric hypertrophic remodeling.

**Figure 1:**
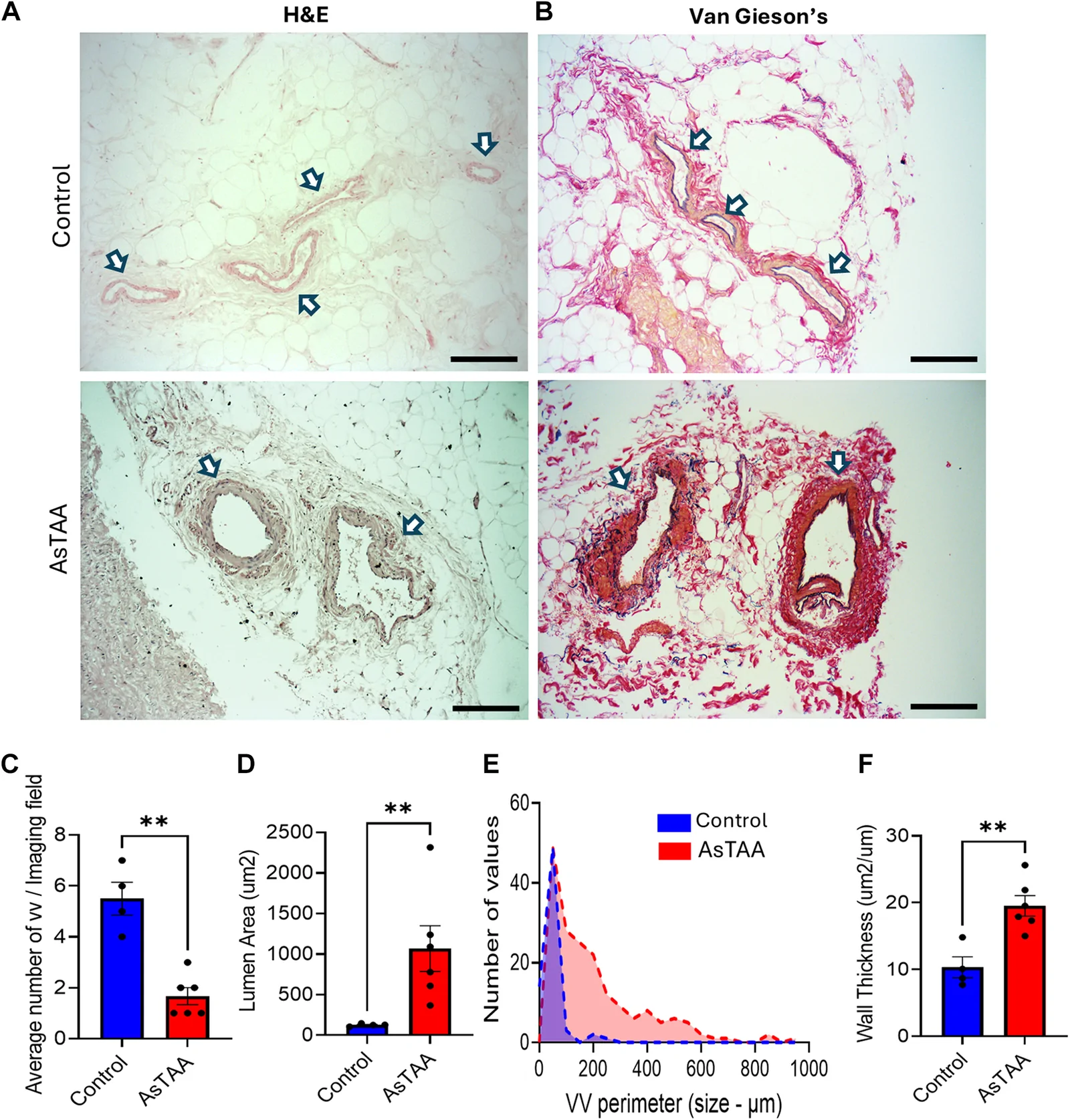
Histological Analysis of Human Adventitia Samples (A) H&E and (B) Van Gieson's stainings. Scale bar = 50 μm. White arrows point to vasa vasorum (VV). Images are representative of n = 1 sample of each group. (C-E) Bar graphs of morphometric measurements for (C) VV density, (D) VV lumen area, (E) VV perimeter size, and (F) VV wall thickness. N = 4 controls and 6 ascending thoracic aortic aneurysms (AsTAA). In (C, D, and F), data are presented as individual values and means ± SEM. In (E), curves show the frequency distribution of the VV perimeter size from all samples in the 2 groups. Analysis by unpaired Mann-Whitney test. ∗∗*P* < 0.01. AsTAA = ascending thoracic aortic aneurysm; H&E = hematoxylin and eosin.

APCs surrounding the VV were identified as PDGFRβ^+^/α-Smooth muscle actin (α-SMA)^-^/CD31^-^ cells (Figures 2A to 2C). IHC analysis revealed a reduction in APC coverage of AsTAA-VV (52.6 ± 3.5%) compared with controls (73.2 ± 2.4%, *P* = 0.001) (Figures 2D to 2F). There was an inverse relationship between VV perimeter and APC coverage; that is, larger VV perimeters corresponded to reduced APC coverage (Figure 2G). Interestingly, whereas control samples showed preserved APC contact with VV, this feature was lost in AsTAA-APCs, which appeared denser in the proper aorta adventitia apart from the VV (*P* = 0.001, Figures 2H and 2I). This suggests that AsTAA-VV rarefaction and enlargement are associated with APC detachment from the original perivascular site.

**Figure 2:**
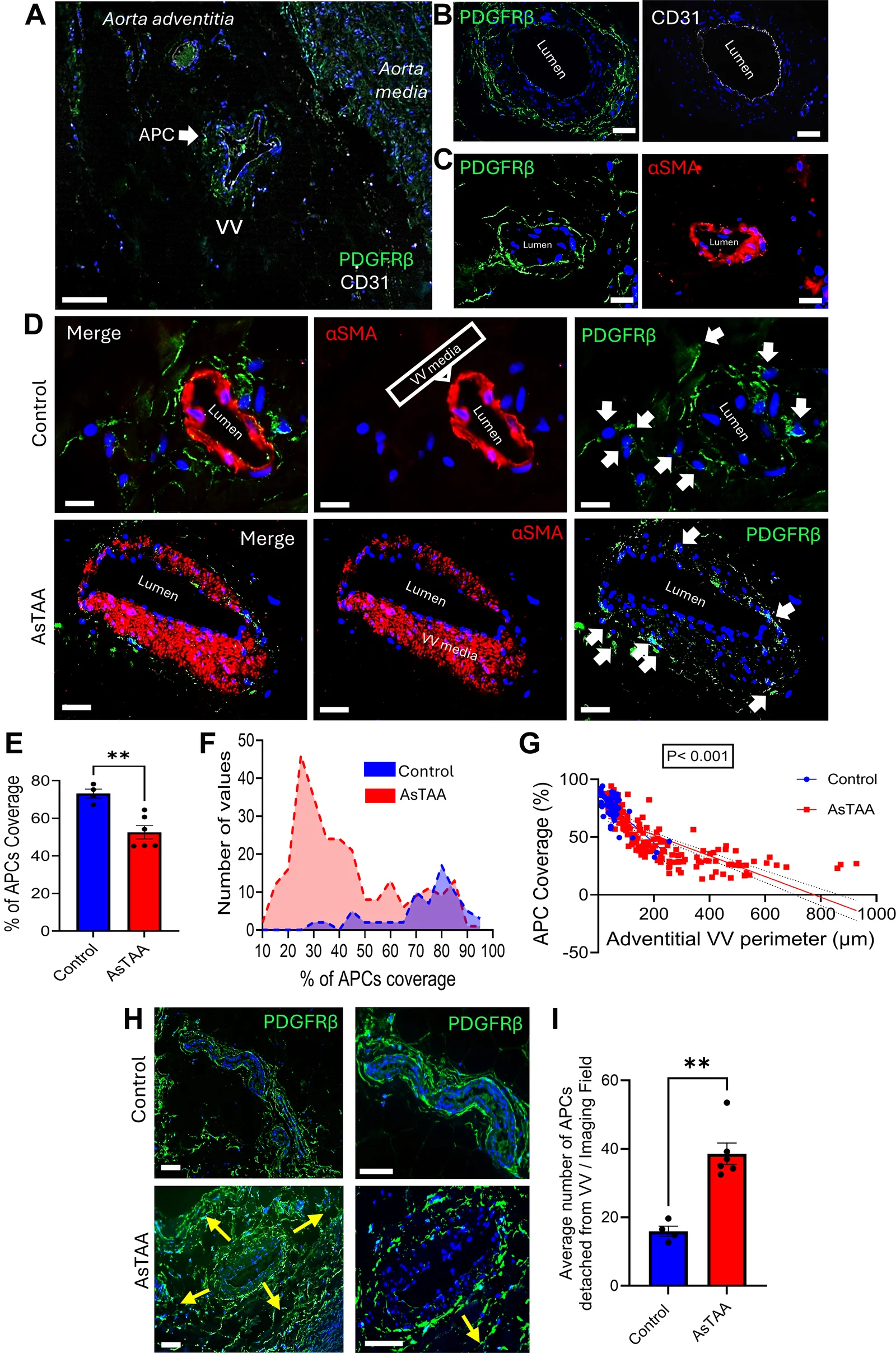
*In situ* Characterization of Adventitial Pericyte-Like Cells (APCs) in Human Aorta Adventitia Using Immunohistochemistry (A) Vasa vasorum (VV)-associated APCs are characterized by expression of platelet-derived growth factor receptor beta (PDGFRβ) and lack of CD31. (B and C) Immunofluorescence images showing examples of VV surrounded by PDGFRβ+ APCs (green). In (B), CD31 labels the VV lumen (white), although in (C), alpha-smooth muscle actin (α-SMA) identifies vascular smooth muscle cells (VSMCs) within the VV tunica media (red). (D) Immunofluorescence images showing coverage of VV with PDGFRβ+ APCs (green, indicated by white arrows) in the 2 groups. α-SMA (red) labels VSMCs. 4’,6-diamidino-2-phenylindole (DAPI) (blue) labels the nuclei. Scale bar = 50 μm. (E) Bar graphs showing a significant decline in APC coverage in ascending thoracic aortic aneurysm (AsTAA) samples compared with controls. N = 4 control and 6 AsTAA. Data are presented as individual values and means ± SEM. Analysis by unpaired Mann-Whitney *U* test. (F) Curves showing the frequency distribution of the percentage of APC coverage of VV from all samples in the 2 groups. (G) Correlation study between the APC coverage and VV perimeter. The study showed a significant difference between the slopes (*P* < 0.001) by a simple linear regression test. (H) Immunofluorescence images showing detachment of APCs (PDGFRβ, green) from VV into the adventitia of AsTAA samples. Yellow arrows show the direction of detachment. DAPI (blue) labels the nuclei. Scale bar = 50 μm. (I) Bar graphs reporting the quantification of APCs distant from VV. N = 4 control and 6 AsTAA. In E and I, data are presented as individual values and means ± SEM. Analysis by unpaired Mann-Whitney *U* test. ∗∗*P* < 0.01.

### APC isolation and antigenic characterization

Using the explant outgrowth method, we isolated APCs from AsTAA and control aorta adventitia samples. Cell sprouting from the adventitial explants was observed after 5-7 days of incubation (Supplemental Figure 1A). The isolated cells exhibited the characteristic morphology of pericytes, appearing as spindle-shaped cells with small central nuclei (Supplemental Figure 1B), consistent with previous descriptions.^22^^,^^39^ Antigenic characterization of sprouting cells at passage zero showed expression of typical pericyte markers PDGFRβ and NG2 and lack of the fibroblast marker PDGFRα and endothelial cell markers CD146 and CD31 (Supplemental Figures 1C and 1D). Moreover, an angiogenesis assay in which isolated APCs were seeded together with AoECs on Matrigel confirmed that APCs physically interact with AoECs and promote the formation and stabilization of endothelial networks, a distinctive feature of pericytes (Supplemental Figure 1E).

Antigenic profiling of expanded cells at P4 revealed no differences between APCs isolated from AsTAA samples and controls. Cells in both groups demonstrated strong positive expression of PDGFRβ and NG2, with negligible expression of CD31, CD146, α-SMA, and PDGFRα (Figures 3A to 3C).

**Figure 3:**
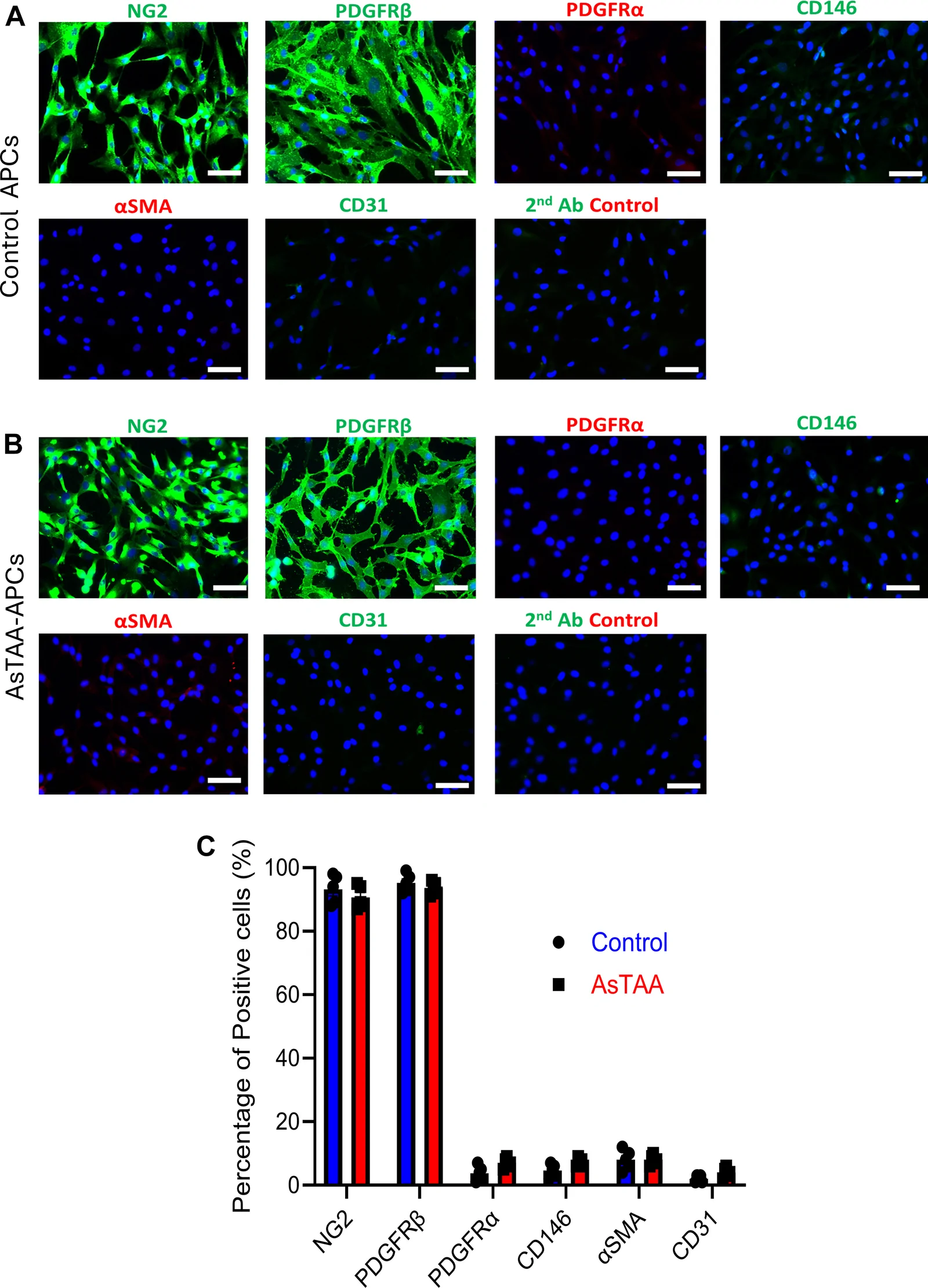
Antigenic Characterization of Isolated Adventitial Pericyte-Like Cells (APCs) (A and B) Immunofluorescence images showing antigenic profile of (A) Control-adventitial pericyte-like cells, and (B) ascending thoracic aortic aneurysm (AsTAA)-APCs at passage 4 of culture. Both groups show strong positivity for pericyte markers neural glial antigen 2 (NG2) and Platelet-derived growth factor receptor beta (PDGFRβ), and negativity for tested endothelial cells, vascular smooth muscle cells, and fibroblast markers. N = 5. Images are representative of 1 sample from each group. 4’,6-diamidino-2-phenylindole (DAPI) labels nuclei in blue. Scale bar = 50 μm. (C) Bar graphs showing the marker quantification. Data are presented as individual values and means ± SEM. N = 5. Expression of each marker between the 2 groups was compared using an unpaired Mann-Whitney *U* test (all not significant).

#### AsTAA-APCs exhibit increased inflammation and disrupted structural gene programs

We used whole-genome transcriptome analysis of control and AsTAA-APCs to explore disease-associated transcriptional features (Figure 4A). Principal component analysis revealed intra-group variability among samples, with only one APC line from each group clearly segregating from the others, whereas 4 samples showed similar transcriptional profiles, indicating that aneurysm status is not a source of significant transcriptomic variation (Figure 4B). This similarity was further supported by hierarchical clustering (Figure 4C). Differential expression analysis identified 50 DEGs (defined as false discovery rate <0.05 and absolute log2 fold change [log2FC] > 1) in AsTAA-APCs compared with controls; this gene set corresponds to 0.3% of the total sequenced transcripts, thereby corroborating that aneurysmatic disease does not significantly affect the APC transcriptome. Of the 50 DEGs, 19 were upregulated and 31 downregulated (Figure 4D). The full list of protein-coding DEGs is reported in the heatmap in Figure 4E (log2FC from +7.9 to −4.6). Among the genes increased in AsTAA-APCs were several inflammatory and immune-associated mediators, including *IL32* (log2FC +2.93)*, TLR2* (+2.81, Toll-Like Receptor 2)*, CXCL6* (+2.37)*, PTPN22* (+2.22, protein tyrosine phosphatase nonreceptor type 22), *CXCL3* (+2.17), and *TNFSF4* (+1.91, TNF superfamily member 4), suggesting activation of proinflammatory pathways. In contrast, genes downregulated in AsTAA-APCs included markers linked to ECM organization, contractile function and cell adhesion, such as *FBLN2* (log2FC −2.19, encoding Fibrillin 2, a component of connective tissue microfibrils involved in elastic fiber assembly)*, EGFLAM* (−2.48, EGF like, fibronectin type III And laminin G domains, which promotes matrix assembly and cell adhesiveness), and *ITGA8* (−4.45, integrin subunit alpha 8, a transmembrane receptor that mediates cell adhesion).

**Figure 4:**
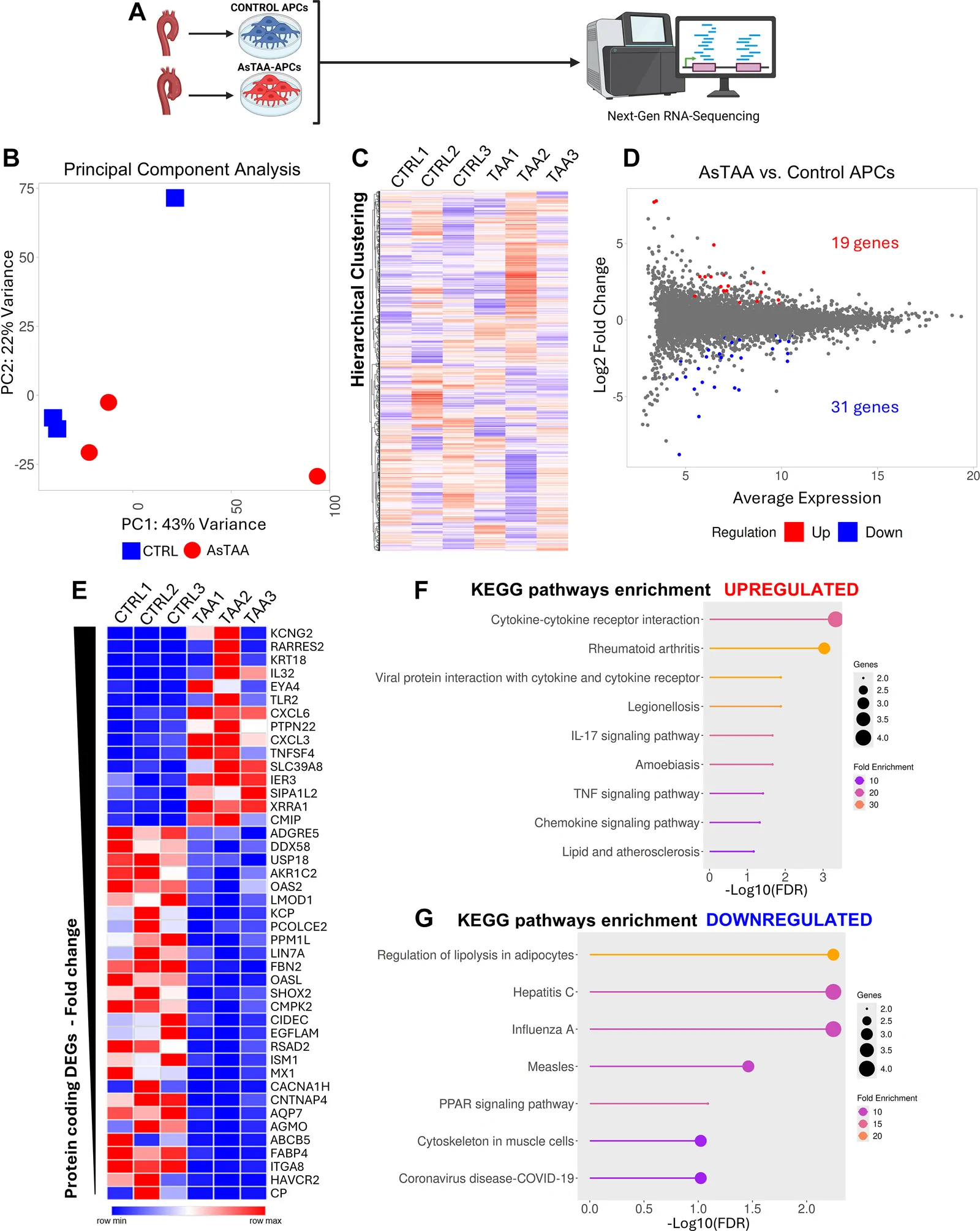
Transcriptomic Analysis of Control and Ascending Thoracic Aortic Aneurysm Adventitial Pericyte-Like Cells (AsTAA APCs) (A) A whole-genome RNA sequencing study was conducted on control and ascending thoracic aortic aneurysm (AsTAA)-derived adventitial pericyte-like cells (APCs) (n = 3/group). (B) Principal component analysis. (C) Hierarchical clustering of the top 10K transcripts. (D) Mean average plot showing that 19 transcripts were significantly upregulated and 31 transcripts downregulated in AsTAA-APCs compared with control cells. Differentially expressed genes (DEGs) were defined as those with a false discovery rate (FDR) < 0.05 and a fold change >2. (E) Heatmap showing all protein-coding DEGs, ranked from highest to lowest fold change. (F and G) Lollipop plots showing enriched Kyoto Encyclopedia of Genes and Genomes (KEGG) pathways based on DEGs. Upregulated pathways are shown in (F), and downregulated pathways are shown in (G). On the y-axis are the Gene Ontology (GO) terms. The x-axis indicates the statistical significance (–Log(10)FDR). The circle size indicates the number of genes involved in each pathway, whereas the lollipop color indicates the enrichment score. IL = interleukins; PPAR = peroxisome proliferator-activated receptors; TNF = tumor necrosis factors.

Pathway enrichment analysis further supported these findings. Upregulated genes in AsTAA-APCs were significantly enriched for pathways related to *Cytokine–cytokine receptor interaction*, *IL-17 signaling*, *TNF signaling*, *Chemokine signaling*, and *Lipid and atherosclerosis* (Figure 4F), consistent with an activated inflammatory phenotype. Conversely, downregulated genes were enriched for the *Cytoskeleton in muscle cells* pathway (Figure 4G), indicating loss of structural functions.

Together, these data indicate that AsTAA-APCs undergo focused transcriptional reprogramming, adopting a phenotype characterized by enhanced inflammatory signaling and suppression of structural gene programs.

### AsTAA-APCs have impaired molecular and functional properties

We next assessed the cell functional and secretory profiles and found that AsTAA-APCs were characterized by altered cellular behavior compared with control cells. Analysis of cell growth revealed that AsTAA-APCs have higher proliferation rates and shorter doubling times (average 72 hours in the AsTAA group vs 106 hours in the control group, *P* < 0.001) (Figure 5A). Additionally, AsTAA-APCs exhibited enhanced motility, as demonstrated by a wound-healing migration assay in which cells were stimulated with PDGF-BB (average 85.3% wound closure after 10 hours vs 58.3% in controls, *P* < 0.001) (Figure 5B). This highly migratory phenotype aligns with the loss of contact between APCs and the VV observed in AsTAA samples and may contribute to the destabilization of the adventitial vascular network.

**Figure 5:**
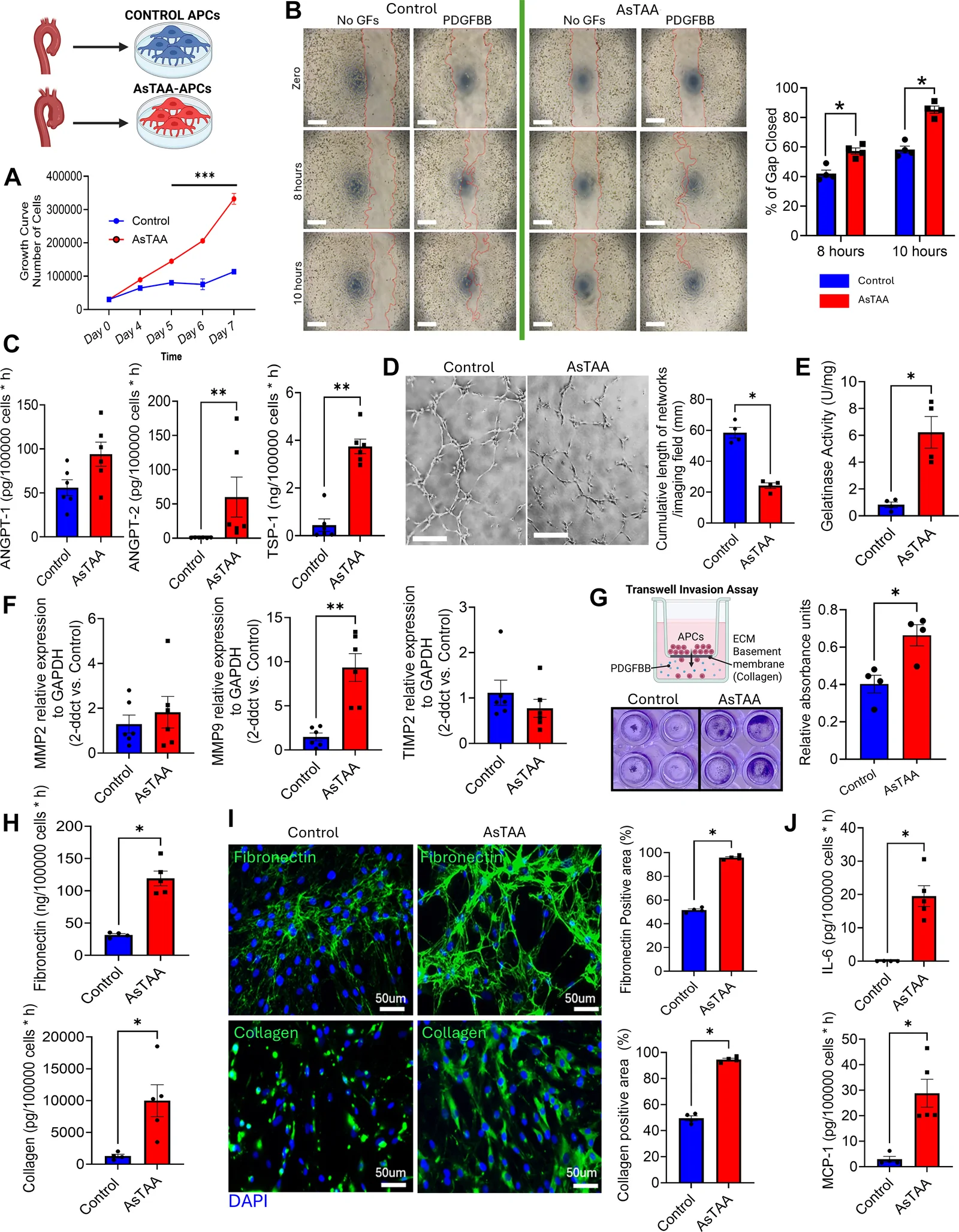
Ascending Thoracic Aortic Aneurysm Adventitial Pericyte-Like Cells (AsTAA-APCs) Show Impaired Functional and Molecular Properties (A) Growth curve showing hyperproliferative status of ascending thoracic aortic aneurysm adventitial pericyte-like cells (AsTAA-APCs) compared with controls. N = 5. Analysis by 2-way analysis of variance. (B) Wound closure migration assay comparing nonstimulated and platelet-derived growth factor BB (PDGFBB)-stimulated cells. Images were snapped after 8 and 10 hours and compared with the initial time zero. Bar graphs report the percentage of wound closure. N = 4. (C) Secreted angiogenic factors in adventitial pericyte-like cell (APC)-conditioned media analyzed by ELISA. N = 6. (D) Angiogenic tube formation assay of APCs cocultured with human aortic ECs (AoECs). Images are representative of 1 sample from each group. N = 4 APCs, n = 1 AoECs. (E) Gelatinase activity fluorescence assay. Results are presented as U/mg. N = 4. (F) Expression of *MMP2*, *MMP9* and tissue inhibitor of metalloproteinases (*TIMP-1*) transcripts as assessed by quantitative reverse transcription polymerase chain reaction. N = 6. (G) Transwell invasion assay testing the APC capacity to migrate through a semipermeable membrane covered by a layer of collagen when attracted by PDGFBB. Migrated cells in the lower chamber were stained with crystal violet (purple) and the color intensity analyzed using a spectrophotometer. The bar graph reports the relative absorbance units. N = 4 for both groups. (H) Secreted extracellular matrix proteins fibronectin and collagen in APC-conditioned media were analyzed using enzyme-linked immunosorbent assay (ELISA). N = 5. AsTAA-APCs and 4 control APCs. (I) Immunofluorescence images and bar graphs showing fibronectin and collagen expression (green fluorescence) in APCs. 4’,6-diamidino-2-phenylindole (DAPI)-labeled nuclei in blue. N = 4 from both groups. (J) Secreted proinflammatory cytokines, interleukin-6 and monocyte chemoattractant protein 1, in APC-conditioned media, analyzed using ELISA. N = 5 AsTAA-APCs and 4 control APCs. All data are presented as individual values and means ± SEM. In (B-J), analysis by unpaired Mann-Whitney *U* test. ∗*P* < 0.05, ∗∗*P* < 0.01 and ∗∗∗*P* < 0.001. ANGPT1 = angiopoietin-1; ECM = extracellular matrix; GFs = growth factors; TSP1 = Thrombospondin-1.

AsTAA-APCs also exhibited an impaired angiogenic profile, evidenced by larger secretion of antiangiogenic factors, including ANGPT-2 and TSP-1, which are known to be associated with vascular regression (*P* = 0.002, Figure 5C). Moreover, in a tube formation assay, AsTAA-APCs showed an impaired ability to form angiogenic networks when in coculture with AoECs on Matrigel (*P* = 0.029, Figure 5D). The diminished angiogenic capacity of AsTAA-APCs represents another hallmark of disrupted vascular homeostasis and repair.

AsTAA-APCs exhibited elevated gelatinase activity (*P* = 0.029, Figure 5E), which was associated with a 5.3-fold increase in *MMP9* gene expression in AsTAA-APCs compared with control-APCs (*P* = 0.002, Figure 5F), indicating enhanced extracellular matrix (ECM) degradation. This feature was further supported by a transwell invasion assay, in which AsTAA-APCs showed a greater capacity than control APCs to degrade and migrate through a thin ECM layer in response to PDGF-BB (*P* = 0.029, Figure 5G). Additionally, AsTAA-APCs secrete higher levels of ECM proteins, including fibronectin and collagen, contributing to abnormal ECM deposition as confirmed by both ELISA (*P* = 0.016, Figure 5H) and ICC (*P* = 0.029, Figure 5I). These changes were accompanied by elevated levels of proinflammatory cytokine IL-6 and MCP-1 (*P* = 0.016) (Figure 5J).

The functional data on cell motility, inflammation, and structural remodeling are all consistent with and validate the transcriptional alterations described previously in AsTAA-APCs.

## Altered AsTAA-APC properties are driven by MEK/ERK signaling and rescued by its inhibition

We next investigated posttranscriptional molecular alterations underlying AsTAA-APC dysfunction. We previously showed that ERK1/2 signaling modulates cardiac pericyte function.^35^ Here, AsTAA-APCs showed increased levels of phosphorylated ERK1/2 (*P* = 0.029), indicating activation of this pathway (Figure 6A). A selective MEKi, PD0325901, successfully prevented ERK1/2 phosphorylation and activation in AsTAA-APCs (Figure 6B) and reduced the nuclear translocation of phosphorylated ERK, a step required for its interaction with nuclear targets (Figure 6C). Conditioning of AsTAA-APCs with PD0325901 for 10 days did not affect cell viability, as assessed by the Calcein/EthD-III assay (Figure 6D). Treated cells showed morphologic changes, including increased cell size and the appearance of intracellular striations (Figure 6E). Additionally, although retaining the expression of pericyte markers PDGFRβ and NG2, PD0325901-treated cells expressed typical VSMC contractile markers, including α-SMA, calponin, smooth muscle myosin heavy chain, smoothelin B, and transgelin (Figure 6F).

**Figure 6:**
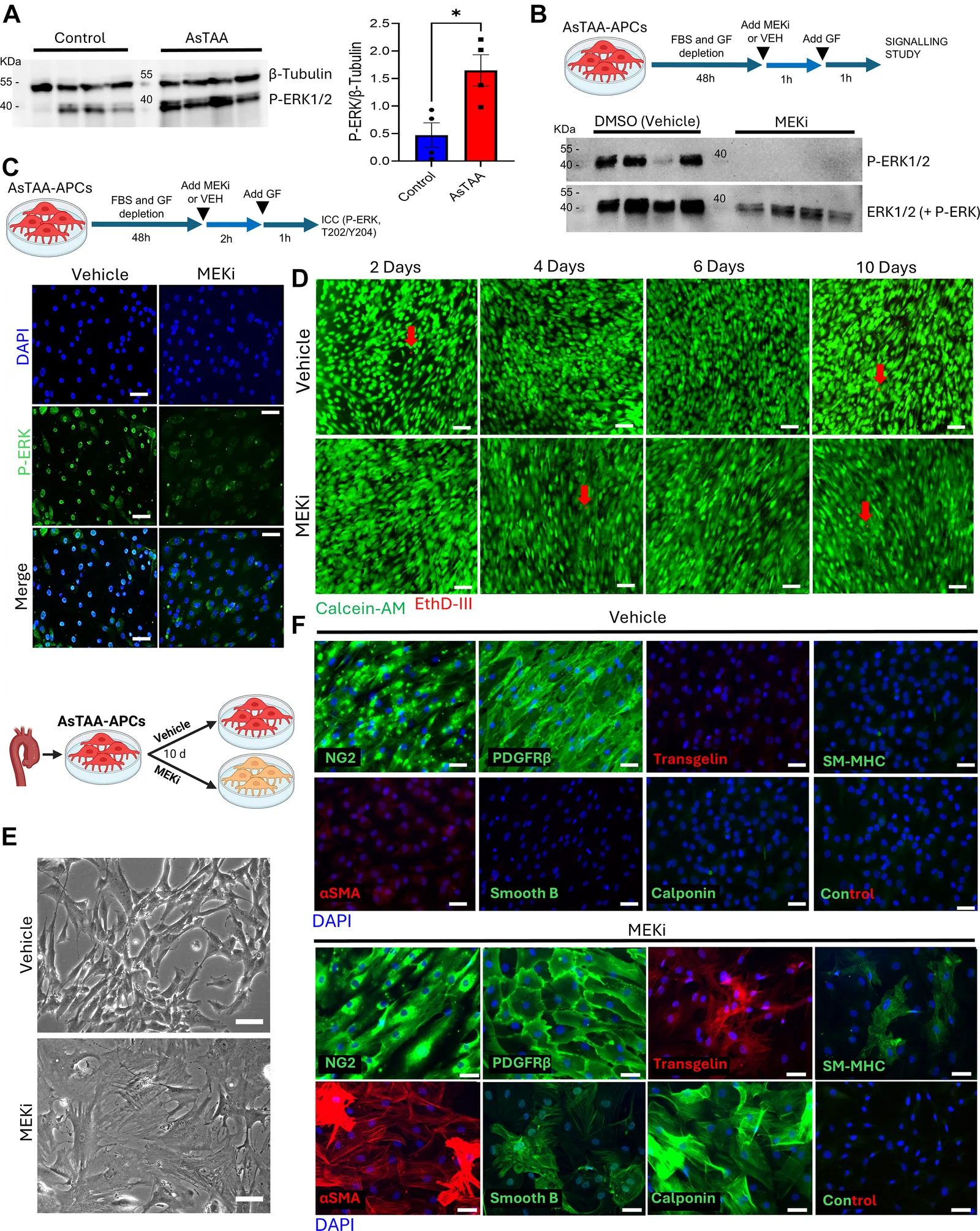
P-ERK Levels in Ascending Thoracic Aortic Aneurysm Adventitial Pericyte-Like Cells (AsTAA-APCs) With or Without PD0325901 Treatment (A) Western blot analysis showing that AsTAA-APCs express higher levels of P-ERK compared with control APCs. N = 4. Data are presented as individual values and means ± SEM. Analysis by unpaired Mann-Whitney *U* test. (B) PD0325901 (MEK inhibitor - MEKi) in a dose of 250 nM successfully inhibited ERK phosphorylation/activation in vitro. N = 4 AsTAA-APCs. (C) Immunofluorescence images showing MEKi treatment inhibited P-ERK (green) nuclear translocation. 4’,6-diamidino-2-phenylindole (DAPI) labels nuclei in blue. N = 2 AsTAA-APCs. Images are representative of 1 sample. In (B and C), APCs were incubated for 48 hours with a growth factor (GF)-free and serum-free medium to switch off molecular signaling, followed by incubation with either the MEKi or its vehicle for 1h, finally followed by stimulation with a GF mix to induce ERK phosphorylation. (D) Viability assay in AsTAA-APCs using Calcein-AM (green, viable cells) and ethidium homodimer III (EthD-III - red, dead cells). N = 3. Images are representative of 1 sample. (E) Brightfield optical images showing morphologic changes of AsTAA-APCs after a 10-day treatment with the MEKi. Cells became larger in size with the appearance of intracellular striations. (F) Immunofluorescence images showing the antigenic profile of AsTAA-APCs 10-day posttreatment. MEKi-treated cells expressed vascular smooth muscle cell markers in addition to pericyte markers. N = 5. Images are representative of 1 sample. Scale bar = 50 μm. DAPI labels nuclei in blue. ∗*P* < 0.05. αSMA = alpha smooth muscle actin; FBS = fetal bovine serum; NG2 = neural-glial antigen 2, PDGFRβ = platelet-derived growth factor receptor beta; SM-MHC = smooth muscle myosin heavy chain.

Compared with the vehicle, PD0325901-treated AsTAA-APC showed suppressed proliferation (*P* = 0.008, Figure 7A). Analysis of the secretome revealed that PD0325901-treated AsTAA-APCs released lower amounts of the anti-angiogenic factor ANGPT-2 (*P* = 0.029, Figure 7B), which was reflected in an improved angiogenic activity of AsTAA-APCs when cocultured with AoECs, as observed in an angiogenic network formation assay (*P* = 0.029, Figures 7C and 7D). Moreover, PD0325901 significantly inhibited AsTAA-APC motility, as shown in the wound-healing migration assay (*P* < 0.001, Figure 7E). Interestingly, PD0325901 treatment also inhibited cell sprouting from adventitial explants (Supplemental Figure 2). Together, these findings demonstrate that the treatment effectively rescues APC aberrations.

**Figure 7:**
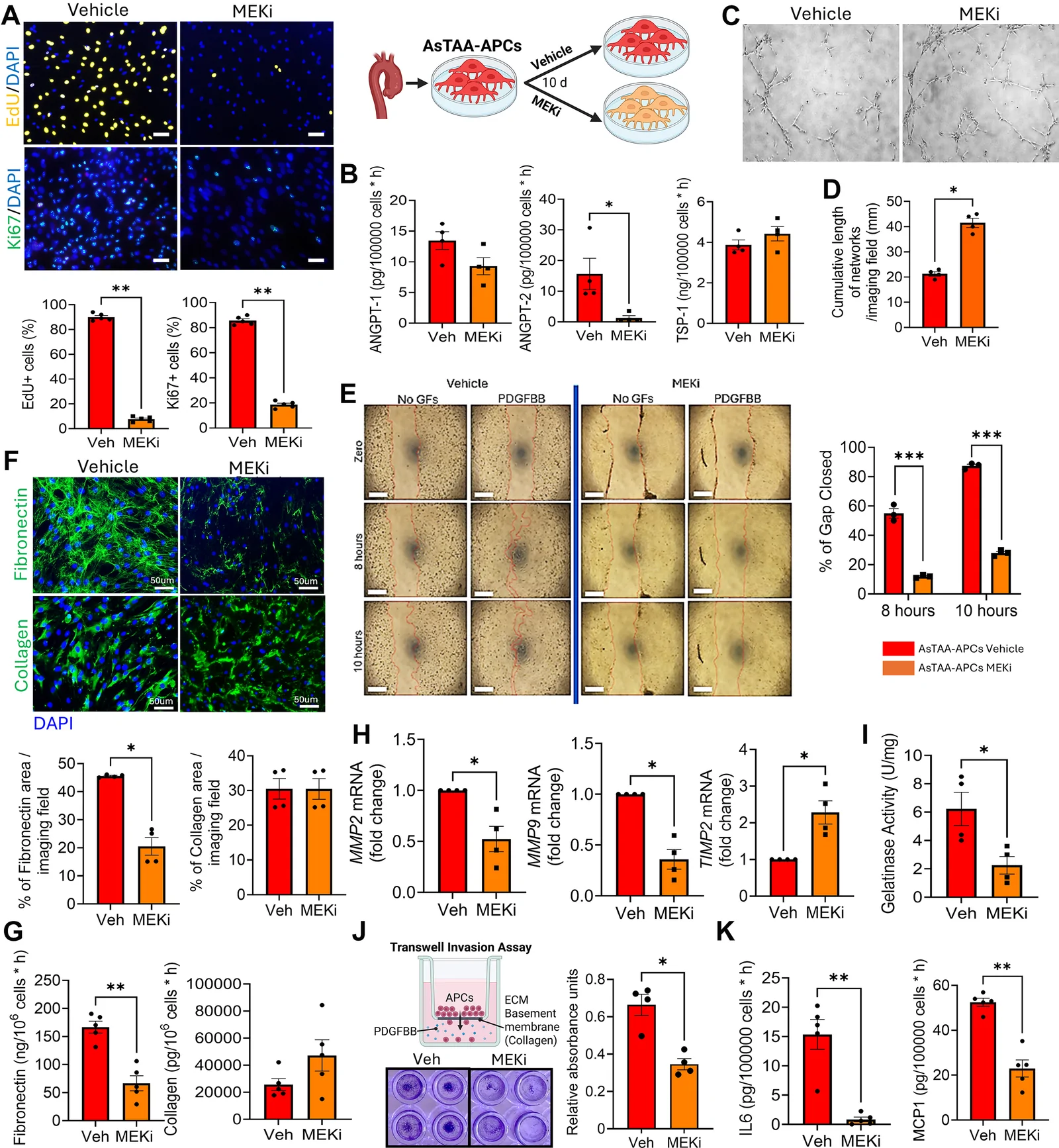
In vitro PD0325901 Treatment Restored Ascending Thoracic Aortic Aneurysm Adventitial Pericyte-Like Cell (AsTAA-APC) Functional and Molecular Properties Comparison of AsTAA-APCs treated for 10 days with either PD0325901 (MEKi) or vehicle. (A) Analysis of proliferating cells using EdU (yellow) and Ki67 (green). DAPI labels nuclei in blue. Images are representative of 1 sample from each group. Scale bar = 50 μm. Bar graphs reporting the percentage of cells positive for EdU and Ki67. N = 5. (B) Secreted angiogenic factors in AsTAA-APC conditioned media measured by enzyme-linked immunosorbent assay (. N = 4. (C and D) Angiogenic tube formation assay with MEKi/Vehicle-treated AsTAA-APCs cocultured with human aortic ECs (AoECs). Images are representative of 1 sample from each group. The bar graph reports the cumulative length of angiogenic networks per imaging field. N = 4 AsTAA-APCs, n = 1 AoECs. (E) Wound closure migration assay comparing nonstimulated and platelet-derived growth factor BB (PDGFBB)-stimulated cells. Images were snapped after 8 and 10 hours and compared with time zero. Bar graphs report the percentage of the wounds closed. N = 3. (F) Immunofluorescence images and bar graphs showing fibronectin and collagen (green fluorescence) expression in AsTAA-APCs. 4’,6-diamidino-2-phenylindole (DAPI) labels nuclei in blue. N = 4. (G) Secreted extracellular matrix proteins fibronectin and collagen in cell-conditioned media. N = 5. (H) Expression of *MMP2*, *MMP9,* and tissue inhibitor of metalloproteinases (*TIMP-1)* transcripts as assessed by quantitative reverse transcription polymerase chain reaction. N = 4. (I) Gelatinase activity fluorescence assay. Results are presented as U/mg. N = 4. (J) Transwell invasion assay testing AsTAA-APC capacity to migrate through a semipermeable membrane covered by a layer of collagen when attracted by PDGFBB. Migrated cells in the lower chamber were stained with Crystal Violet (purple) and the color intensity analyzed using a spectrophotometer. The bar graph reports the relative absorbance units. N = 4. (K) Secreted proinflammatory cytokines interleukin 6 and monocyte chemoattractant protein 1 in AsTAA-APC conditioned media. N = 5. All data are presented as individual values and means ± SEM. Analysis by unpaired Mann-Whitney *U* test. ∗*P* < 0.05, ∗∗*P* < 0.01 and ∗∗∗*P* < 0.001.

Furthermore, PD0325901-treated cells produced lower amounts of fibronectin, as assessed using ICC (*P* = 0.029, Figure 7F) and ELISA (*P* = 0.008, Figure 7G). Conversely, treatment did not affect collagen synthesis. PD0325901 conditioning reduced *MMP2* and *MMP9* transcripts in AsTAA-APCs, along with increased expression of tissue inhibitor of metalloproteinases 2 (*TIMP-2)* mRNA (*P* = 0.029, Figure 7H). Importantly, PD0325901-treated AsTAA-APCs showed reduced gelatinase activity (*P* = 0.029, Figure 7I), which was reflected in a reduced cell capacity to invade a layer of ECM in an in vitro invasion assay (Figure 7J). Finally, MEK inhibition reduced the levels of the proinflammatory cytokines MCP-1 and IL-6 in the culture media of AsTAA-APCs, consistent with a dampening of the inflammatory response. (*P* = 0.008, Figure 7K).

Collectively, these findings suggest that PD0325901 can effectively restore the altered properties of AsTAA-APCs by downregulating the MEK/ERK signaling pathway.

### PD0325901 conditioning shifts AsTAA-APCs towards a vascular-protective phenotype

Next, we used whole-genome transcriptomics to explore in greater depth the mechanisms by which MEK inhibition shifts AsTAA-APCs toward a phenotype that promotes vascular integrity and stability (Figure 8A). Principal component analysis showed that PD0325901-treated AsTAA-APCs formed a distinct cluster from vehicle-treated cells and shifted away from the untreated aneurysmal profile, indicating that MEK inhibition significantly reprogrammed the cell transcriptome (Figure 8B). This difference was confirmed by hierarchical clustering (Figure 8C).

**Figure 8:**
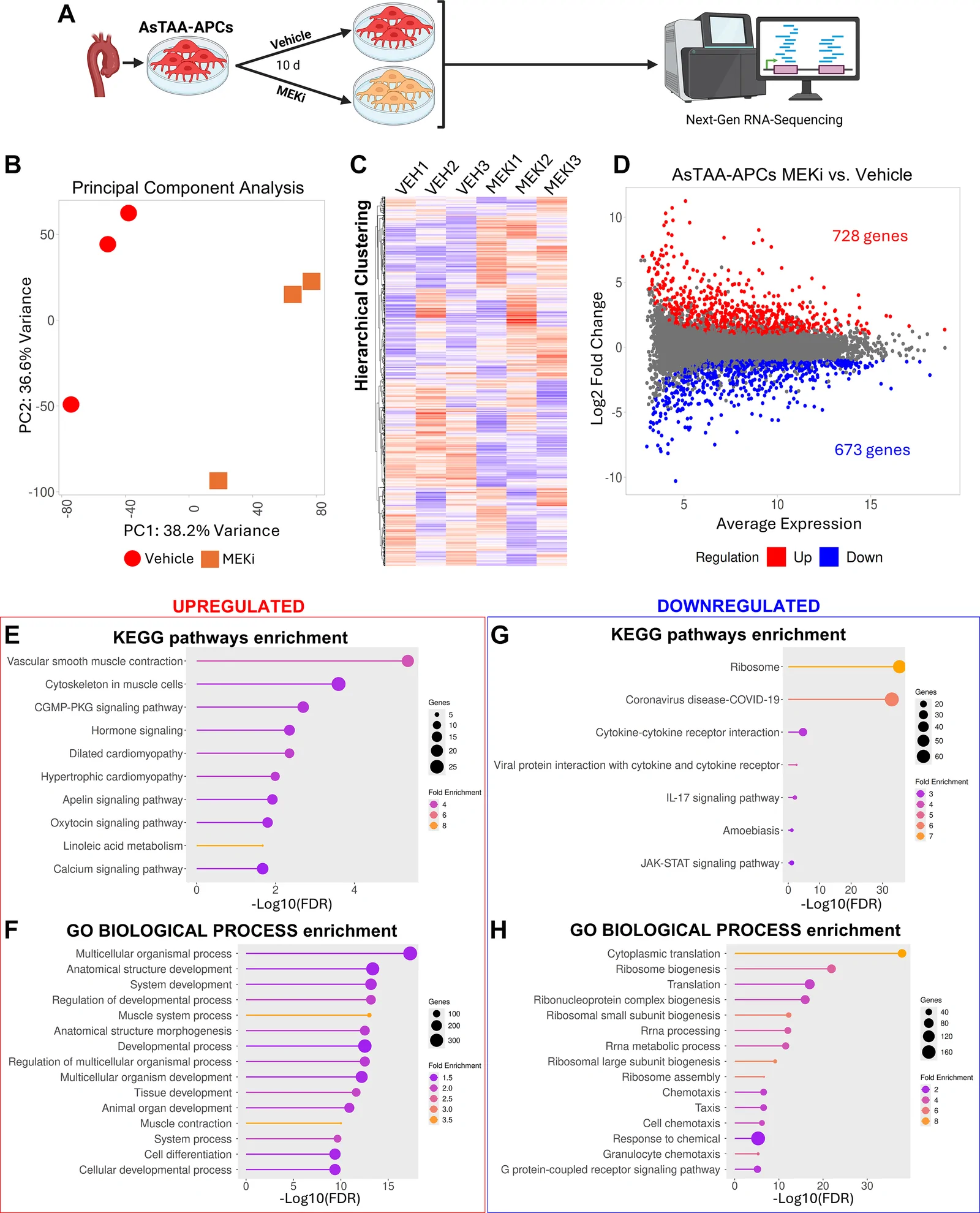

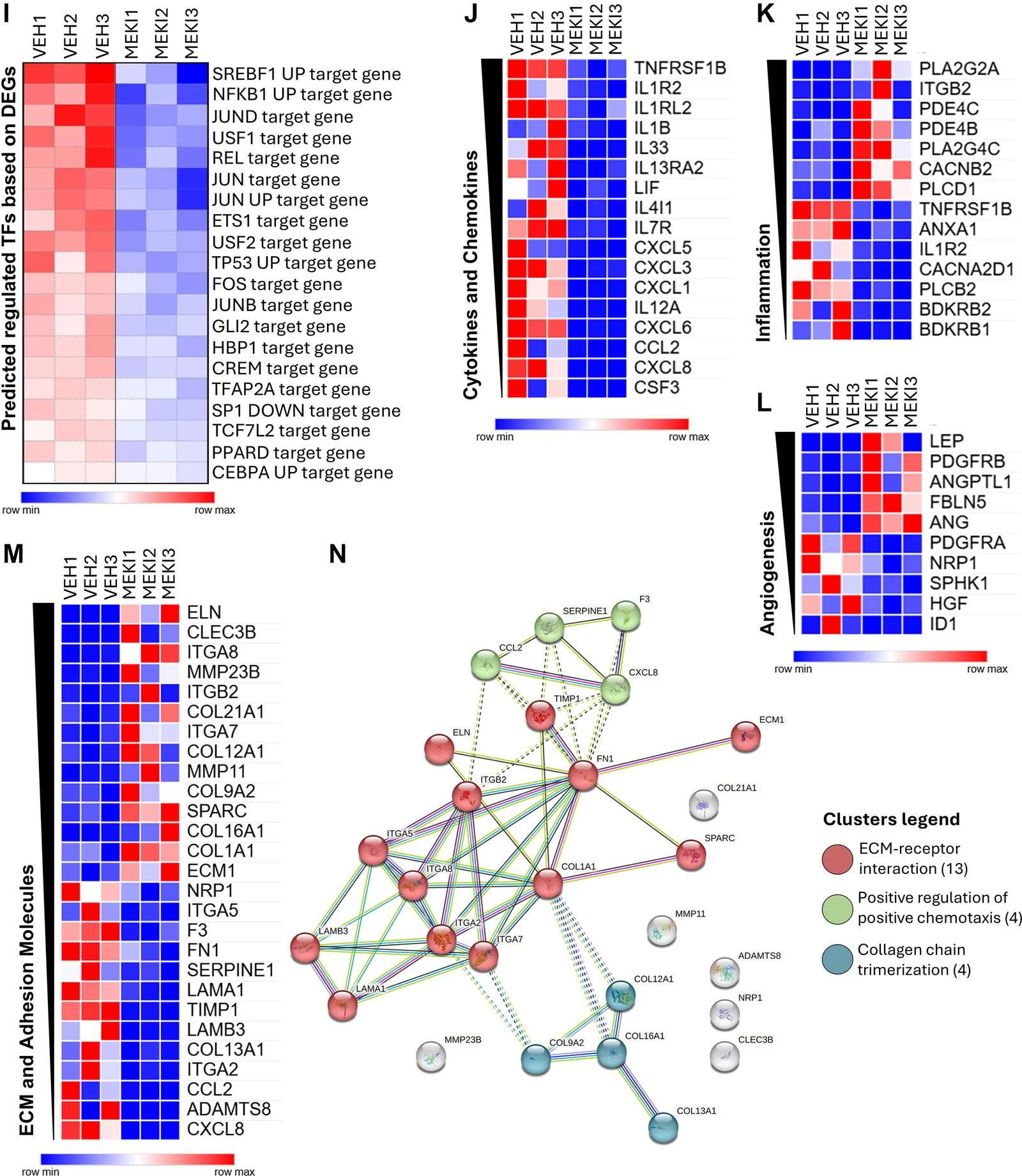
Transcriptomic Analysis of Ascending Thoracic Aortic Aneurysm Adventitial Pericyte-Like Cells (AsTAA-APCs) Conditioned With the MEKi or Vehicle (A) A whole-genome RNA-Sequencing study was conducted on AsTAA-APCs conditioned for 10 days with mitogen-activated protein kinase kinase inhibitor (MEKi) (PD0325901) or its vehicle, dimethyl sulfoxide (DMSO) (n = 3). (B) Principal component analysis. (C) Hierarchical clustering of the top 10K transcripts. (D) Mean average plot showing 728 transcripts significantly upregulated and 673 downregulated in MEKi-conditioned AsTAA-APCs compared with vehicle-treated cells. Differentially expressed genes (DEGs) were defined as those with a false discovery rate (FDR) < 0.05 and a fold change >2. (E-H) Lollipop plots showing enriched Kyoto Encyclopedia of Genes and Genomes (KEGG) and biological process pathways based on DEGs. Upregulated pathways are shown in (E and F), and downregulated pathways are shown in (G and H). On the y-axis are the Gene Ontology (GO) terms. The x-axis indicates the statistical significance (–Log(10)FDR). The circle size indicates the number of genes involved in each pathway, whereas the lollipop color indicates the enrichment score. (I) Prediction of regulated transcription factors (TFs) based on DEGs using the TFactS R package. Genes targeted by TFs, such as JUN, FOS, and ETS—which are all activated by ERK1/2—are downregulated in MEKi-treated AsTAA-APCs, corroborating the suppression of extracellular signal-regulated kinase 1/2(ERK1/2) downstream molecular signaling. (J-M) Heatmaps reporting DEGs associated with cytokines and chemokines (J), inflammation (K), angiogenesis (L), extracellular matrix and adhesion molecules (M). All genes are ranked by log2 fold change. (N) Transcripts in (M) were analyzed using STRING to identify protein-protein interaction networks. Clusters emerging from the analysis of GO Biological process terms are indicated in different colors.

Differential expression analysis identified 728 transcripts significantly upregulated and 673 downregulated in MEKi-treated AsTAA-APCs compared with vehicle-treated cells (Figure 8D). Gene set enrichment analysis further indicated that PD0325901 upregulated pathways associated with *Vascular smooth muscle contraction*, *Cytoskeleton in muscle cells*, and *Muscle system* (Figures 8E and 8F). Downregulated pathways encompass *Cytokine-cytokine receptor interaction*, *IL17 signaling*, and *Cell chemotaxis* (Figures 8G and 8H).

Prediction of regulated TFs revealed that DEGs controlled by TFs such as Jun proto-oncogene, Fos proto-oncogene, and ETS proto-oncogene 1, all downstream targets of ERK1/2 signaling, were significantly downregulated in MEKi-treated AsTAA-APCs, corroborating effective suppression of ERK1/2-mediated transcriptional activity (Figure 8I).

MEKi treatment reduced the expression of transcripts encoding cytokines, chemokines, and inflammatory mediators in AsTAA-APCs (Figures 8J and 8K). In particular, *CXCL3* and *CXCL6*, which were upregulated in AsTAA-vs control- APCs (log2FC +2.17 and + 2.37, respectively), were markedly downregulated by the MEKi in AsTAA-APCs (log2FC −4.34 and −5.22) (Figure 8J). MEK inhibition upregulated angiogenesis-related transcripts, including *LEP (leptin,* log2FC +7.71), *PDGFRB* (+2.44), *ANGPL1* (Angiopoietin-Related Protein 1, +2.09), and *ANG* (angiogenin, +1.28) (Figure 8L). *FBLN5* (Fibulin 5, +1.57), a secreted ECM protein playing a role both in vascular remodeling and elastic fiber formation, was also induced by the MEKi (Figure 8L). Furthermore, MEKi conditioning promoted a broad transcriptional reprogramming of genes encoding ECM and adhesion molecules (Figure 8M). Among the most regulated are *ELN* (elastin, log2FC +8.25), *CLEC3B* (C-Type Lectin Domain Family 3 Member B, +5.93, which enables calcium ion binding activity), and *ITGA8* (+4.86, integrin mediating cell adhesion, which was downregulated in AsTAA-vs control- APCs (−4.45)). Several collagen transcripts were also upregulated (*COL21A1, COL12A1, COL9A2, COL16A1, COL1A1*, log2FC from +2.76 to +1.35). Interestingly, MEKi treatment induced *SPARC* (secreted protein acidic and cysteine-rich, log2FC +1.62), encoding a matricellular protein that we demonstrated is secreted by vascular pericytes and plays a critical role in stabilizing ECM remodeling postinjury.^46^ Among the most downregulated transcripts we found *ADMTS8* (ADAM metallopeptidase with thrombospondin type 1 motif 8, log2FC −6.58, a disintegrin and metalloproteinase enzyme). STRING (version 12.0) network analysis of the transcripts encoding ECM and adhesion molecules evidenced a strong biological connection (high confidence interaction score of 0.7 and protein-protein interaction [PPI] enrichment *P* value < 1 × 10^−16^) and identified 3 main clusters related to *ECM-receptor interaction*, *Positive regulation of positive chemotaxis*, and *Collagen chain trimerization* (Figure 8N).

Together, these findings provide mechanistic evidence that pharmacologic inhibition of MEK/ERK signaling induces substantial transcriptional reprogramming in AsTAA-APCs and rescues the altered functional phenotype. By suppressing inflammatory and matrix-disruptive pathways whereas restoring gene programs associated with ECM stabilization, vascular homeostasis, and cell adhesion, PD0325901 shifts AsTAA-APCs toward a more homeostatic phenotype. This transition may contribute to preserving the integrity of the adventitial vascular niche and the surrounding aortic architecture, potentially helping to limit aneurysm progression.

### PD0325901 treatment halts aneurysm progression and preserves aortic wall integrity in a mouse model

An in vivo study explored the effects of PD0325901 treatment in a C57BL/6J mouse nonatherosclerotic model of AsTAA. After 72 hours of subcutaneous implantation of ANG-II minipumps, mice were given oral MEKi PD0325901 (10 mg/kg/d) or vehicle (DMSO), mixed with sugar-free strawberry jelly, daily for 14 days (Figure 9A). Healthy mice not undergoing any treatment were used as controls. The effective inhibition of ERK1/2 phosphorylation in the aorta of the PD0325901 group was confirmed using western blotting (Figure 9B).

**Figure 9:**
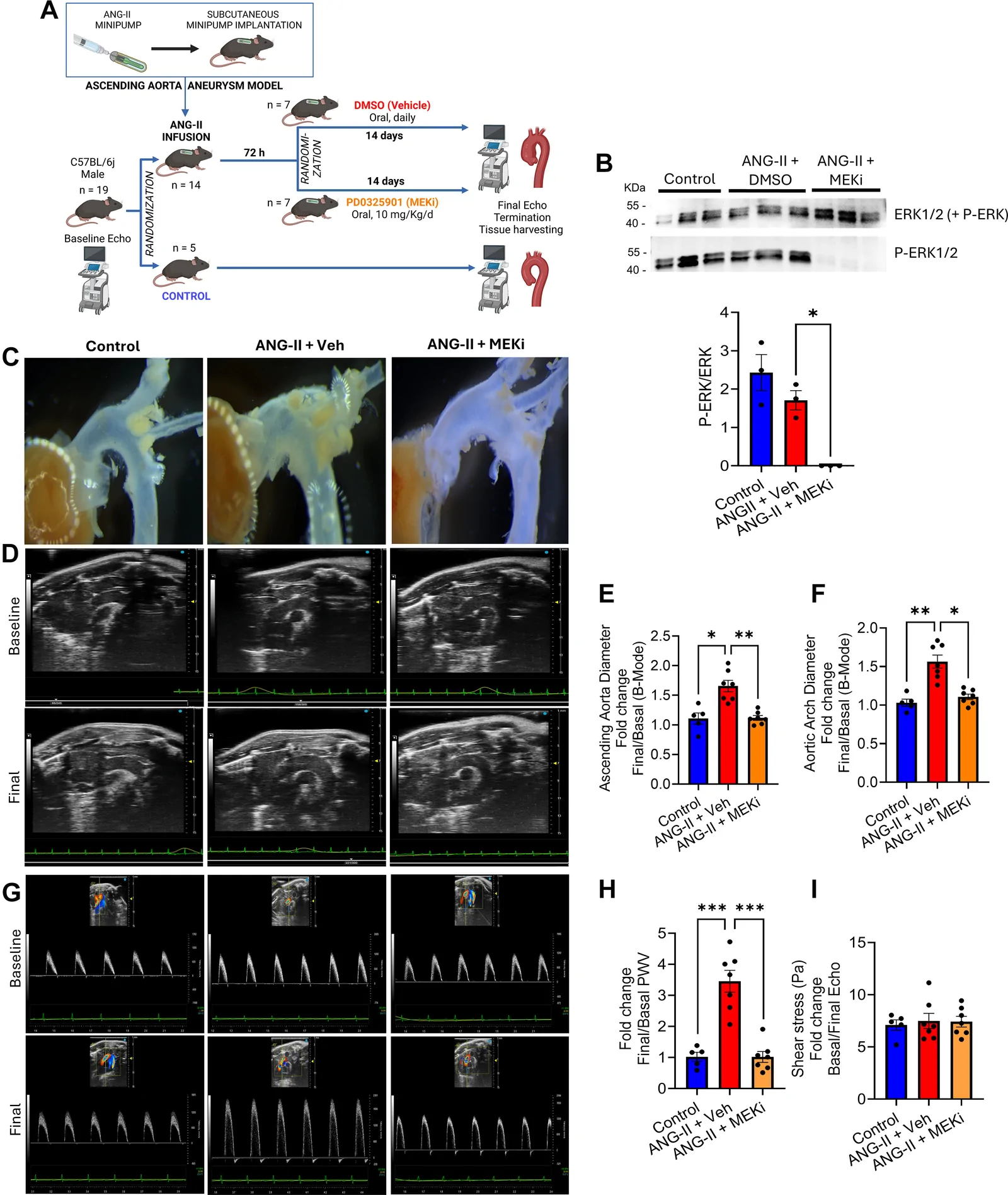
Oral PD0325901 Treatment for 14 Days Halted Thoracic Aortic Aneurysm (TAA) Progression in a Murine Model (A) Cartoon showing the experimental protocol. Mice were started on oral PD0325901 (10 mg/kg/d) or vehicle (dimethyl sulfoxide, DMSO) embedded in jelly 72 hours post-Angiotensin II (ANG-II) minipump implantation and followed up for 14 days. Control mice, included as a reference, did not receive any treatment. (B) Western blot showing protein expression for total extracellular signal-regulated kinase 1/2 (ERK1/2) and P-ERK in aorta lysates. N = 3 per group. (C) Images acquired using a stereo microscope (Leica) showing the mouse aortas after harvesting. (D) Images of baseline and final echocardiography showing the ascending aorta and proximal arch. Images representative of 1 sample from each group. (E and F) Inner edge-to-edge diameters were presented as a fold change of the measurements at the final time point against basal timepoints. (G) Pulse wave Doppler images of baseline and final echocardiography representative of 1 sample per group. (H) Pulse wave velocity (PWV) was calculated using the equation (d-d0)/(T2-T1), where (d-d0) = distance, T1 = start time, and T2 = end time.^76^ PWV calculations were presented as final/basal fold change. (I) Oral PD0325901 treatment has no effect on aorta shear stress. Equation used is (wall shear stress (WSS) = 4 ∗ 0.0035 ∗ mean velocity (m/s)/diameter (m)), where (0.0035 Pa s) is the standard mouse blood viscosity.^77^ In (E, F, H, and I) N = 5 control, n = 7 vehicle and mitogen-activated protein kinase inhibitor (MEKi). All data are presented as individual values and means ± SEM. The unpaired Mann-Whitney *U* test was used to compare group pairs as indicated. ∗*P* < 0.05, ∗∗*P* < 0.01 and ∗∗∗*P* < 0.001.

As previously reported,^43^^,^^44^ ANG-II treatment induced a dilatation of the ascending aorta and aortic arch compared with untreated controls (Figure 9C), although none of these mice developed AAA. At the 14-day follow-up, PD0325901-treated mice exhibited reduced dilatation of the ascending and arch aortas compared with vehicle-treated mice (Figure 9C). In the vehicle group, there was an absolute increase in the inner diameter of the ascending aorta from the basal to the final echo of 0.69 ± 0.06 mm (*P* = 0.014), compared with 0.16 ± 0.11 mm in the treated group (*P* = 0.003) (Figures 9D and 9E). Likewise, the aortic arch diameter increased by 0.57 ± 0.02 mm from baseline to final echo in the vehicle group (*P* = 0.002), compared with only 0.13 ± 0.01 mm in the MEKi group (*P* = 0.010) (Figure 9D and 9F). Doppler analysis revealed that pulsed wave velocity (PWV), an arterial stiffness index clinically used for risk stratification of TAA, was significantly lower in MEKi-treated mice (with a final-to-basal difference of −2.1 ± 63.23 cm/s) compared with the vehicle group (+497 ± 116 cm/s, *P* < 0.001) (Figures 9G and 9H). Shear stress remained unchanged in all groups (Figure 9I). All acquired echocardiography indices are reported in Supplemental Table 7. Importantly, no mortality was documented, suggesting the treatment was safe.

Histologic analyses of aortic samples showed that PD0325901, when compared with vehicle, preserved the aortic medial area (*P* = 0.039, Figure 10A) and elastin content (*P* = 0.020, Figure 10B) and inhibited collagen deposition and fibrosis (*P* < 0.001, Figure 10C) in TAA mice, which is in line with reduced stiffness highlighted by the PWV data. Importantly, MEK inhibition reduced the levels of the active form of MMP9 in the tunica media (*P* = 0.018; Figure 10D), consistent with a potential role in preserving structural integrity. Moreover, IHC analysis showed that oral PD0325901 treatment significantly increased the expression of the VSMC marker α-SMA in the media (*P* < 0.001, Figure 10E). In the adventitia, compared with the vehicle, MEKi significantly reduced CD45+ inflammatory cell content (*P* = 0.024, Figure 10F) as well as adventitial cell apoptosis (*P* < 0.001, Figure 10G) with no effect on adventitial PDGFRβ+ cell density (Figure 10H).

**Figure 10:**
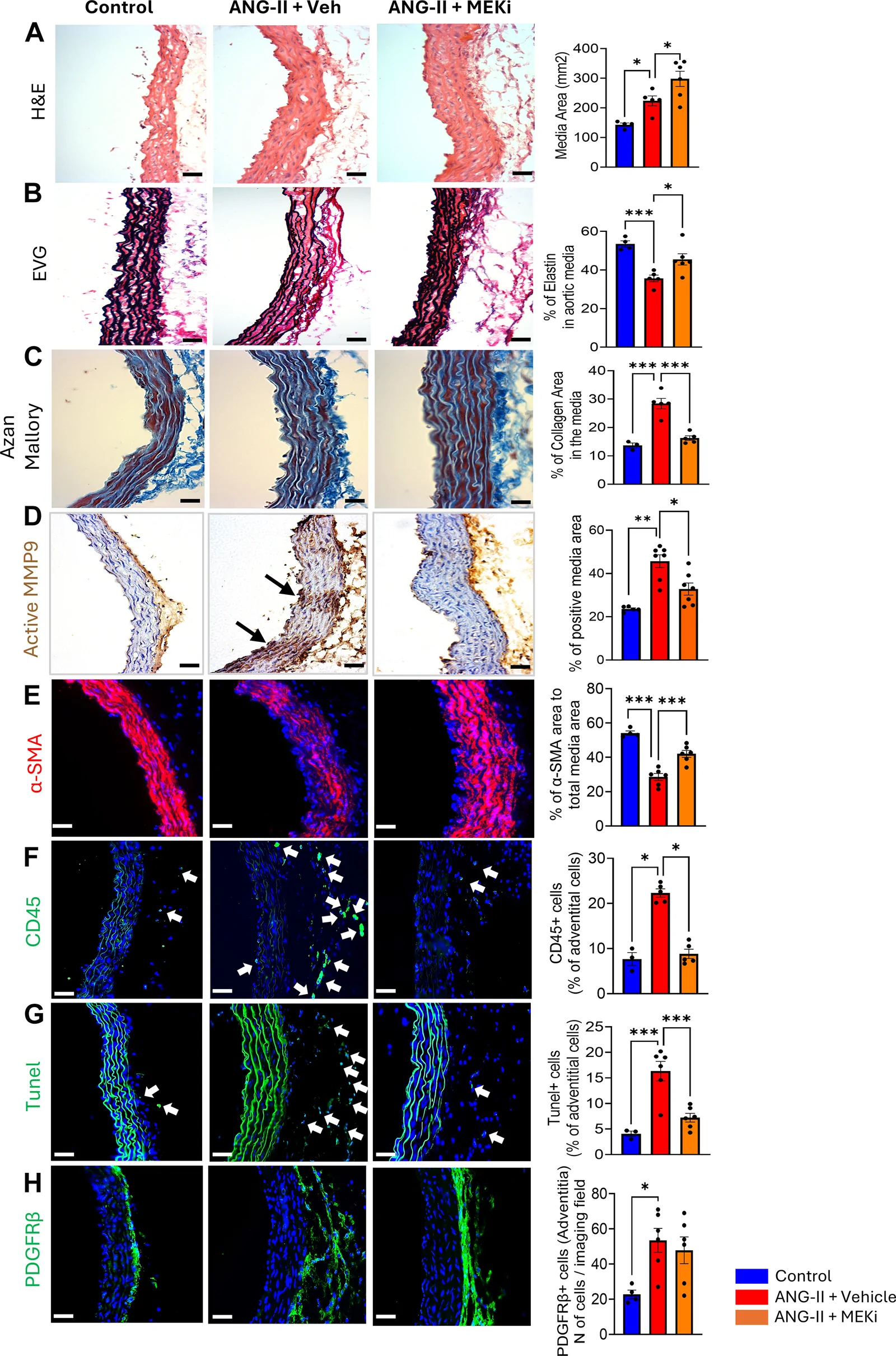
PD0325901 Treatment Inhibits Angiotensin-II (ANG-II)-Induced Aortic Remodeling (A) Hematoxylin and eosin (H&E) images and bar graphs showing aortic medial area. (B) Van Gieson (EVG) elastin staining images and bar graphs showing elastin medial content. (C) Azan Mallory images and bar graphs showing medial collagen content. (D) Immunohistochemistry (IHC) staining showing active MMP9 expression (brown, 3,3′-diaminobenzidine). Bar graphs report the expression of active MMP9 limited to the tunica media. (E) IHC images and bar graphs showing expression of α-Smooth muscle actin (α-SMA - red fluorescence) in medial vascular smooth muscle cells. (F) IHC images and bar graphs showing recruited CD45+ cells (green). Arrows point to positive cells. (G) Fluorescence images and bar graphs showing apoptotic adventitial cells (Tunel+, in green). Arrows point to positive nuclei. (H) IHC images and bar graphs showing pericytes (platelet-derived growth factor receptor beta [PDGFRβ]+ cells) in the adventitia. In all fluorescent images, 4’,6-diamidino-2-phenylindole (DAPI) was used to stain nuclei (blue). All images are representative of 1 sample from each group. Scale bar = 50 μm. Data are presented as individual values and means ± SEM. The unpaired Mann-Whitney *U* test was used to compare group pairs as indicated. ∗*P* < 0.05, ∗∗*P* < 0.01 and ∗∗∗*P* < 0.001.

In summary, oral PD0325901 treatment halted the progression of TAA formation, with significant benefits to the integrity of the adventitial niche and improvements in the typical hallmarks of inflammation and stiffness.

## Discussion

Our study shows that AsTAAs are characterized by pericyte-like cells with a dysfunctional phenotype, altered spatial organization, and activation of MEK/ERK signaling. We also found that the selective MEKi PD0325901 can restore AsTAA-APC function in vitro and halt aneurysm progression in an in vivo model of nonatherosclerotic TAA. Although further work is needed to confirm the role of APCs in vivo, these findings point to a plausible mechanism (illustrated in Figure 11) through which dysfunctional APCs may contribute to disease progression.

**Figure 11:**
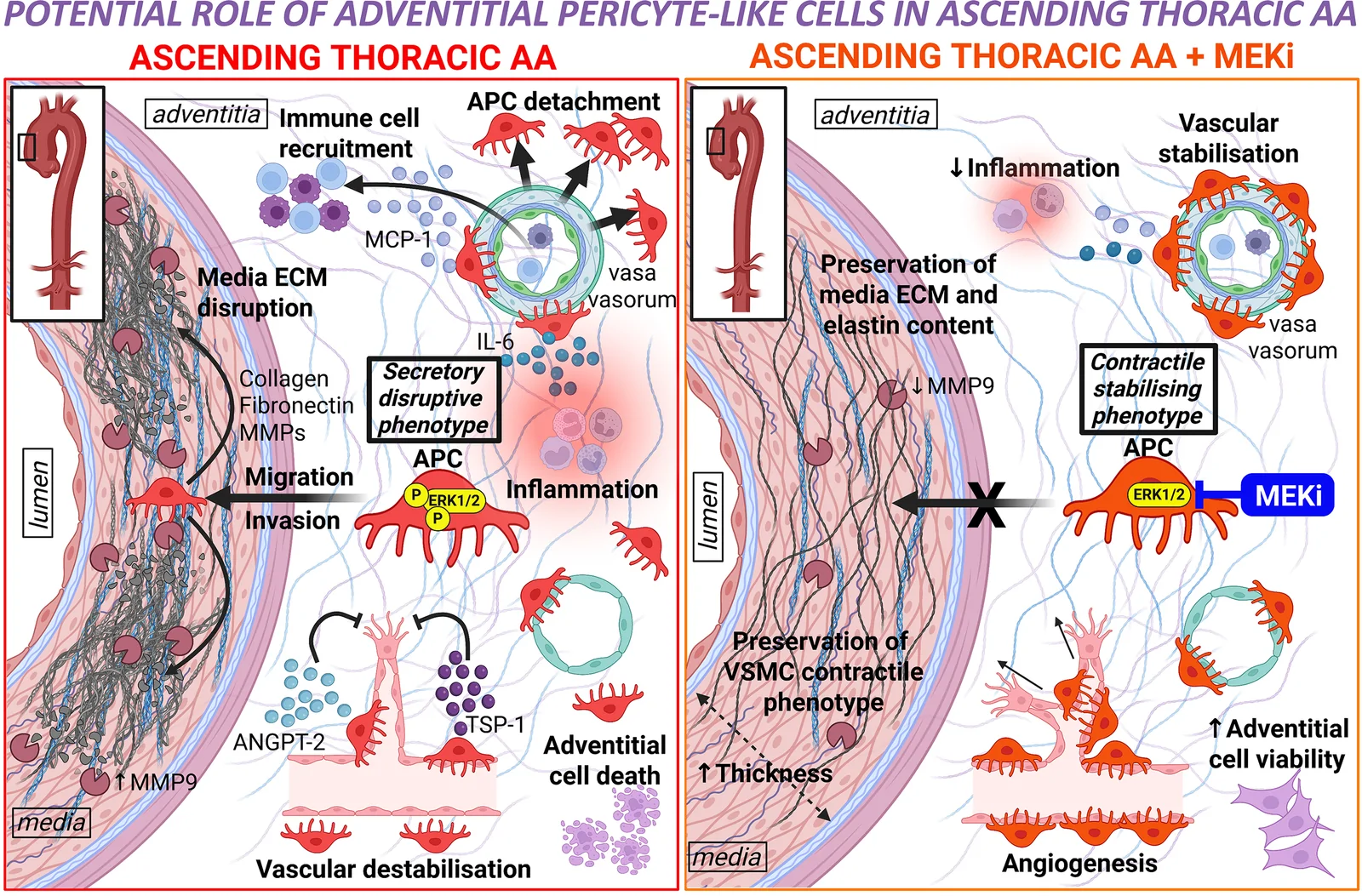
Schematic Illustration Summarizing the Key Findings From Both the Human Patient and Mouse Studies The cartoon illustrates the potential adventitial pericyte-like cells (APC)-driven mechanism in ascending thoracic aortic aneurysm (TAA) pathogenesis (left) and the beneficial effects of MEK inhibition therapy (MEKi - PD0325901) in rescuing APC function, preventing aortic wall remodeling, and halting TAA progression (right). Although further investigation is needed to confirm the role of APCs in TAA pathogenesis and progression in vivo, our in vitro findings strongly suggest that APCs are key players in TAA.

Created in BioRender.com.

Because the human analysis was performed on end-stage AsTAAs, the observed disruption of the adventitial vasculogenic niche may reflect a late feature consequential to the accumulating damage during disease progression. At the same time, it remains possible—though not yet proven—that early disruption of VV–APC interactions may contribute to aneurysm development by impairing key adventitial functions such as perfusion, ECM stability, and immune surveillance.

Histologic analyses of human AsTAA samples by Billaud et al revealed notable morphometric alterations in the adventitial VV, characterized by reduced density and enlarged vessels with wider lumens and thicker walls.^15^ Our findings are in line with these observations and show a marked reduction (around 30%) in VV-associated PDGFRβ^+^ APC coverage compared with control aortas. This decrease was inversely correlated with VV perimeter as shown in the results as well as with lumen diameter and wall thickness (data not shown), suggesting a link between APC loss and VV instability and fragility.

Interestingly, we also observed that AsTAA-APCs are more abundant in regions distant from the VV, consistent with the idea that these cells may detach and migrate into the surrounding adventitia. This interpretation aligns with previous studies reporting an increased density of adventitial progenitor cells in the outer media of patients with TAAs, pointing toward a more invasive cellular behavior.^47^^,^^48^ As an alternative or complementary explanation, breakdown of the ECM and basement membrane may disrupt the structural support that normally anchors pericytes. In parallel, aortic wall dilation and edema could increase physical separation, whereas inflammation and hypoxia may further compromise pericyte–endothelial interactions.

Pericytes are essential in regulating endothelial cell stability, vascular tone, and maintaining microvascular integrity.^49^ The observed loss of VV-APC coverage in AsTAA samples mirrors findings in other vascular pathologies, where pericyte loss/failure and detachment are associated with increased vascular permeability, hypoxia, and inflammation.^50^, ^51^, ^52^ In AsTAAs, the increased expression of ANGPT-2 and TSP-1 points to a pathologic phenotype that may promote destabilization of adventitial vascularization.

Isolated APCs displayed typical pericyte markers (PDGFRβ and NG2) while lacking endothelial and VSMC markers.^22^ Interestingly, there were no antigenic differences between control- and AsTAA-APCs. RNA sequencing revealed that control and AsTAA-APCs share highly similar transcriptional profiles, with only ∼0.3% of transcripts showing significant differences between the 2 study groups. Taken together, these findings suggest that the phenotypic changes observed in AsTAA-APCs are largely functional and may be driven by posttranscriptional mechanisms, likely involving dysregulated intracellular signaling pathways such as MEK/ERK.^53^^,^^54^

Proliferative and migratory alterations have been reported in tumoral pericytes, contributing to cancer metastasis.^55^ Proteolytic degradation of the ECM is a shared hallmark of both cancer and TAAs, leading to the breakdown of structural barriers that normally constrain cell migration. Here we report that AsTAA-APCs contribute to ECM degradation, as evidenced by their ability to degrade and invade a thin ECM layer—an effect likely driven by elevated gelatinase activity and increased *MMP-9* gene expression. Together with dysregulated synthesis of ECM components, this process weakens the structural integrity of the aortic wall, making it more vulnerable to hemodynamic stress and progressive dilatation. In addition, RNA sequencing revealed activation of proinflammatory pathways in AsTAA-APCs. The increased secretion of cytokines such as MCP-1 and IL-6, observed in vitro, may sustain a self-perpetuating inflammatory loop, promoting immune cell recruitment to the adventitia and further destabilizing the vascular microenvironment.^44^^,^^56^

As key regulators of vascular stability, pericytes typically facilitate angiogenesis and maintain endothelial cell integrity.^57^^,^^58^ The inability of AsTAA-APCs to form stable networks with AoECs, associated with disrupted secretion of angiogenic factors, suggests a diminished homeostatic turnover of VV and a weakened reparative response to injury through angiogenesis. In turn, reduced VV density in aneurysm tissue impairs oxygen and nutrient supply to the outer layers of the aorta.^15^ Hypoxia within the adventitia can further stimulate the formation of larger and more fragile VVs, similar to what is observed in aggressive tumors.^15^^,^^16^

Anticancer drug repurposing is attracting much attention for the treatment of cardiovascular disease, the underpinning rationale residing in shared molecular and cellular features between the 2 diseases.^31^^,^^59^ And more specifically, clinical studies showed a higher coincidence of aortic aneurysm and cancers, which was independent of other cardiovascular diseases and traditional risk factors.^60^^,^^61^ PD0325901 is a second-generation small-molecule inhibitor with specific activity against MEK1/2, exhibiting enhanced potency and fewer side effects compared with first-generation compounds. PD0325901 has been tested in several tumors, including colon cancer, breast cancer, non–small cell lung cancer, and melanoma, and was well tolerated by patients in phase I-II trials.^62^ MEK/ERK pathway hyperactivation is also a known TAA hallmark.^28^^,^^29^

Our findings point to APCs as a potential target of PD0325901 therapy. Whole-genome RNA sequencing showed substantial transcriptional reprogramming in AsTAA-APCs following treatment. PD0325901 dampened inflammatory and matrix-remodeling pathways while restoring gene programs linked to ECM stabilization, vascular homeostasis, and cell adhesion. At a functional level, the drug shifted AsTAA-APCs from a synthetic toward a more contractile phenotype, improved their angiogenic capacity, reduced their inflammatory profile, and helped rebalance their secretome and matricellular output. In parallel, by enhancing APC adhesion and limiting their migration and invasion into the surrounding matrix, MEK inhibition may support the preservation of the adventitial vascular niche. Overall, these results suggest that the broad effects of MEK inhibition could help strengthen the aortic wall and potentially slow its dilatation.

To challenge this theory, we tested PD0325901 therapy in a mouse TAA model induced by the vasoconstrictor ANG-II,^43^^,^^44^ a potent inducer of MEK/ERK, JNK, and p38.^63^^,^^64^ We chose the ANG-II model because it more closely represents sporadic, nonsyndromic, nonatherosclerotic AsTAAs. Results confirm a prior study that subcutaneous ANG-II (1,000 ng/kg/min) preferentially generates TAAs regardless of lipid status.^43^ Other researchers have addressed the MEK/ERK pathway in TAA models of Marfan syndrome,^65^^,^^66^ which are less suitable for mimicking the clinical condition of interest than the ANG-II model.

Our findings indicate that PD0325901 can limit AsTAA progression and improve aortic wall integrity in a model of sporadic thoracic aortic aneurysm. The treatment appears to support both the structural and functional properties of the aortic wall through inhibition of the MEK/ERK pathway, a key signaling driver in aneurysm development.^28^^,^^29^ Echocardiographic observations of ascending aorta and aortic arch diameters demonstrate that PD0325901 reduces aneurysmal dilatation. The treatment also reduces PWV, a strong indicator of wall stiffness.^67^^,^^68^ The result indicates improved aortic compliance, which is confirmed by histologic data. For instance, enhanced α-SMA expression in the media indicates VSMC stability, whereas increased elastin content, reduced fibrosis, and reduced active MMP9 expression indicate restoration of ECM integrity. PD0325901 may prevent pathologic medial remodeling and aortic wall thinning, which are hallmarks of TAA development and progression.^69^^,^^70^ The reduction in adventitial inflammation and apoptosis is therapeutically relevant because persistent inflammation and cell death contribute to the development of TAA.^44^^,^^71^^,^^72^ The lack of a substantial difference in adventitia PDGFRβ+ cell density between the vehicle and MEKi groups may be because of the oral MEKi treatment starting at 72 hours post-ANG-II infusion.

Efficacy was accompanied by encouraging safety outcomes; however, longer-term studies are warranted to exclude potential adverse effects associated with this anticancer drug. Implantable endovascular devices may also offer a promising route for targeted delivery of MEK inhibitors, though the limited time of drug release may limit this approach. Platforms such as the Aortyx patch or the Gore Excluder TAMBE illustrate how localized, catheter-based interventions can directly access the diseased aortic segment.^73^ Adapting these technologies to incorporate controlled drug release could enable site-specific delivery of MEK inhibition, potentially reducing systemic exposure and associated side effects compared with conventional administration. At the same time, such an approach would need to carefully address the duration and kinetics of drug release to ensure sustained therapeutic activity without compromising safety or device integration.

## Conclusions

Our findings suggest that PD0325901 effectively modulates pathologic MEK/ERK signaling in APCs, restoring their physiologic phenotype. This therapeutic approach has the potential to halt aneurysm growth by preserving the critical structural and functional properties of the aortic wall, particularly the adventitial niche, thereby opening up promising perspectives for future treatments.

Epidemiologic studies have reported a nonrandom association between cancer and aortic aneurysms, with coexistence rates ranging from approximately 1%-17% and evidence of increased cancer risk in aneurysm patients.^60^^,^^74^^,^^75^ Given this bidirectional association, along with the increasing incidental detection of cancer during imaging for AsTAA, patients with both conditions may represent a suitable group in which to initially evaluate MEKi therapy. These agents target pathways relevant to both diseases and are already used in oncology, where their safety profile is relatively well characterized. In this context, potential side effects may be more acceptable, whereas in patients without cancer, the risk–benefit balance would need to be considered more cautiously.

### Study limitations

Characterization of the VV and APCs in late-stage human AsTAA was performed using surgical specimens. However, early-stage samples—when surgery is not indicated—were not accessible, limiting our ability to investigate the cellular and molecular evolution of the disease. As a result, it remains unclear whether APC dysfunction represents an early and persistent driver of disease development or a consequence of advanced-stage pathology.

Because of interspecies differences, the translational relevance of our findings should be interpreted with caution. Analysis of the vasa vasorum and APCs in the animal model was also constrained by the relative scarcity of VV in the mouse aorta. It is therefore possible that the mechanisms underlying the therapeutic effects of MEK inhibition in mice—athough not excluding a direct effect on pericytes—primarily involve other cellular compartments or layers of the aortic wall. Further studies are needed to evaluate the long-term effects of PD0325901 in AsTAAs, particularly by comparing APC-related effects with its potential protective actions on resident vascular cells and recruited immune cells. Additional pharmacokinetics and safety studies will also be important for refining the MEK inhibition regimen, assessing potential long-term adverse effects, and ruling out unwanted interactions with standard cardiovascular therapies.

Perspectives **COMPETENCIES IN MEDICAL KNOWLEDGE**: AsTAA is a complex, life-threatening condition involving all 3 layers of the aortic wall. Although much attention has traditionally been focused on medial degeneration and intimal changes, the adventitia emerges as an active, dynamic contributor to disease progression. Within the adventitia lies the perivascular niche surrounding the VV, rich in APCs that play critical roles in maintaining vessel wall integrity, modulating ECM turnover, and regulating inflammation.

Our study revealed that the MEK/ERK signaling pathway is a central regulator of APC behavior. Under extracellular stimuli, such as chronic inflammation and angiotensin II–induced stress, this pathway becomes overactivated, leading to APC dysfunction characterized by excessive migration, impaired angiogenesis, and secretion of proinflammatory and matrix-degrading factors. These changes weaken the adventitial support system, ultimately compromising aortic wall structure and promoting aneurysm expansion.

By selectively inhibiting MEK1/2 with PD0325901, we were able to pharmacologically reprogram APCs toward a more quiescent and reparative phenotype. This not only stabilized the aortic wall, preserving elastin, reducing fibrosis, and maintaining compliance, but also suppressed inflammatory cell infiltration and adventitial apoptosis. This strategy, although not a substitute for surgery in advanced cases, may offer a critical window of stability in early or moderate AsTAA stages, potentially delaying the need for urgent intervention and allowing patients to reach elective surgery in a more controlled, safer state.

**TRANSLATIONAL OUTLOOK**: Patients with AsTAA often remain asymptomatic until the point of life-threatening dissection or rupture, underscoring the urgent need for medical therapies that can prevent disease progression before surgical thresholds are reached. Our findings demonstrate that MEK pathway overactivation contributes to APC dysfunction and adventitial remodeling, leading to aortic wall weakening. Importantly, pharmacologic MEK inhibition with PD0325901 reversed these pathologic processes, reduced aneurysm growth, and preserved vascular compliance in vivo. These results support a paradigm in which early pharmacologic targeting of APC dysfunction could stabilize aneurysmal disease before the onset of fatal complications. The identification of APC reprogramming as a therapeutic mechanism introduces a novel translational strategy that may complement current imaging-based surveillance, particularly for patients in the gray zone who are not yet surgical candidates.

### Data Availability

All the data that support the findings of this study are included in the manuscript and are available from the corresponding authors upon request. The RNA-Sequencing dataset is available in NCBI's Gene Expression Omnibus under accession GSE328194.

## Funding Support and Author Disclosures

This work was funded by the Ministry of Higher Education of the Arabic Republic of Egypt (MM24/21, PhD scholarship to Khaled AK Mohammed) and by the British Heart Foundation (FS/IBSRF/23/25173, Intermediate Basic Science Research Fellowship to Dr Elisa Avolio). The authors have reported that they have no relationships relevant to the contents of this paper to disclose.
