# Supplementary material for: Targeting the ERK Signaling Pathway to Halt Adventitia Remodeling of Ascending Thoracic Aortic Aneurysm
Source: JACC Basic Transl Sci. 2026 Sep 25;11(10):101665. doi: 10.1016/j.jacbts.2026.101665 (PMC13635404; doi:10.1016/j.jacbts.2026.101665)
Supplement: Supplementary file 1 [file mmc1.docx]

**Targeting the ERK signaling pathway to halt adventitia remodeling of ascending thoracic aortic aneurysm**

**SUPPLEMENTAL MATERIAL**

**Suppl. Figure 1. Isolation and antigenic characterization of APCs. (A)** Explant outgrowth method. Adventitia samples were cut into small pieces, cultured in tissue culture flasks with full EGM2 media and incubated in an incubator (37 C, 5% CO2). Cells started to sprout after 5-7 days, pieces were removed, and cells expanded. **(B)** Brightfield optical image of sprouting cells at passage 1, showing morphology of pericytes (i.e., spindle shape with small central nuclei). **(C)** Immunofluorescence images showing the antigenic profile of the sprouting cells at passage zero. N = 4. Images are representative of 1 sample. **(C)** Bar graph showing the quantitative analysis of the various markers in APCs. Data are presented as individual values and means ± SEM. N = 4. **(E)** Images representative of angiogenic assays in which APCs and aortic endothelial cells (AoECs) were seeded either alone or together on Matrigel. APCs were labelled with a red fluorescent cell tracker. The images in the panel below clearly show the physical interaction between APCs and AoECs. APCs promote the formation and stabilization of AoEC tubular networks.
