# Supplementary material for: Targeting the ERK Signaling Pathway to Halt Adventitia Remodeling of Ascending Thoracic Aortic Aneurysm
Source: JACC Basic Transl Sci. 2026 Sep 25;11(10):101665. doi: 10.1016/j.jacbts.2026.101665 (PMC13635404; doi:10.1016/j.jacbts.2026.101665)
Supplement: Supplementary file 2 [file mmc2.docx]

**Targeting the ERK signaling pathway to halt adventitia remodeling of ascending thoracic aortic aneurysm**

**SUPPLEMENTAL MATERIAL**

**Suppl. Figure 2. Ex vivo study to assess the effect of PD0325901 on APC sprouting from adventitia explants.** Brightfield optical images showing cell sprouting from explants incubated with the MEKi (250 nM) or Vehicle (DMSO) for 10 days. MEKi significantly inhibited APC sprouting and migration from tissue. N = 4. Images are representative of 1 sample. Data are presented as individual values and means+/-SEM. Analysis by unpaired Mann-Whitney U test. **P*<0.05.
