# Supplementary material for: Targeting the ERK Signaling Pathway to Halt Adventitia Remodeling of Ascending Thoracic Aortic Aneurysm
Source: JACC Basic Transl Sci. 2026 Sep 25;11(10):101665. doi: 10.1016/j.jacbts.2026.101665 (PMC13635404; doi:10.1016/j.jacbts.2026.101665)
Supplement: Supplementary file 3 [file mmc3.docx]

**Targeting the ERK signaling pathway to halt adventitia remodeling of ascending thoracic aortic aneurysm**

**SUPPLEMENTAL MATERIAL**

**Suppl. Table 1.** **Summary of the clinical data of controls and TAA patients**. Values are presented as the mean ± SEM or n (%). ***P < 0.001. Group comparisons were performed using unpaired Student’s t-test for continuous variables and Fisher’s exact test for categorical variables.

| **Factor** | **Control** | **Aortic Aneurysm** |
| --- | --- | --- |
| **N** | 6 | 9 |
| **Male sex** | 4 (66.7%) | 5 (55.6%) |
| **Age (years)** | 62 ± 1.7 | 65 ± 2.7 |
| **Asc. Aorta Diameter (cm)** | 2.1 ± 0.13 | 5.8 ± 0.12 *** |
| **HTN** | 6 (100.0%) | 8 (88.9%) |
| **Diabetes** | 3 (50.0%) | 4 (44.4%) |
| **Statins** | 4 (66.7%) | 2 (22.2%) |
| **ACE-inhibitor** | 5 (83.3%) | 7 (77.8%) |
| **ARB** | 1 (16.7%) | 1 (11.1%) |
