# Supplementary material for: Targeting the ERK Signaling Pathway to Halt Adventitia Remodeling of Ascending Thoracic Aortic Aneurysm
Source: JACC Basic Transl Sci. 2026 Sep 25;11(10):101665. doi: 10.1016/j.jacbts.2026.101665 (PMC13635404; doi:10.1016/j.jacbts.2026.101665)
Supplement: Supplementary file 4 [file mmc4.docx]

**Targeting the ERK signaling pathway to halt adventitia remodeling of ascending thoracic aortic aneurysm**

**SUPPLEMENTAL MATERIAL**

**Suppl. Table 2.** **Human samples used in the manuscript.** List of experiments performed with the aorta adventitia samples and extracted APCs in the two experimental groups.

| **Experimental group** | **Adventitial Sample ID** | **Experiments performed with tissue samples and/or extracted APCs** |
| --- | --- | --- |
|  | **Control 1** | Histology, IHC, ICC, Doubling time, Migration, ELISA, Angiogenesis assay, Gelatinase activity, RT-qPCR, WB, RNA-Seq, Invasion assay |
|  | **Control 2** | Histology, IHC, ICC, RT-qPCR, ELISA, Doubling time, Migration, RNA-Seq, Invasion assay |
|  | **Control 3** | Histology, IHC, ICC, Angiogenesis assay, RT-qPCR, WB, ELISA, Gelatinase activity, WB, RNA-Seq |
|  | **Control 4** | ICC, RT-qPCR, Angiogenesis assay, Doubling time, Migration, ELISA, Invasion assay |
|  | **Control 5** | ICC, RT-qPCR, WB, Gelatinase activity, Doubling time, ELISA, Invasion assay |
|  | **Control 6** | Histology, IHC, RT-qPCR, Doubling time, ELISA, Migration, WB, Angiogenesis assay, Gelatinase activity |
|  | **AsTAA 1** | Histology, IHC, ICC, Migration, ELISA, Angiogenesis assay, Gelatinase activity, RT-qPCR, WB |
|  | **AsTAA 2** | Histology, IHC, ICC, RT-qPCR, ELISA, Doubling time, Migration, in-vitro MEKi, Invasion assay |
|  | **AsTAA 3** | Histology, IHC, ICC, Angiogenesis assay, RT-qPCR, WB, ELISA, Gelatinase activity, WB, in-vitro MEKi, RNA-Seq |
|  | **AsTAA 4** | Histology, IHC, ICC, RT-qPCR, Angiogenesis assay, Doubling time, Migration, ELISA, in-vitro MEKi |
|  | **AsTAA 5** | ICC, RT-qPCR, WB, Gelatinase activity, Doubling time, ELISA, Invasion assay |
|  | **AsTAA 6** | Histology, IHC, RT-qPCR, Doubling time, ELISA, WB, Angiogenesis assay, Gelatinase activity, in-vitro MEKi, RNA-Seq |
|  | **AsTAA 7** | ICC, ELISA, Angiogenesis assay, Gelatinase activity, RT-qPCR, in-vitro MEKi, RNA-Seq, Invasion assay |
|  | **AsTAA 8** | ICC, Migration, ELISA, Angiogenesis assay, RT-qPCR, Invasion assay |
|  | **AsTAA 9** | Histology, IHC, ICC, Migration, Doubling time, Angiogenesis assay, RT-qPCR. |
