# Supplementary material for: Targeting the ERK Signaling Pathway to Halt Adventitia Remodeling of Ascending Thoracic Aortic Aneurysm
Source: JACC Basic Transl Sci. 2026 Sep 25;11(10):101665. doi: 10.1016/j.jacbts.2026.101665 (PMC13635404; doi:10.1016/j.jacbts.2026.101665)
Supplement: Supplementary file 5 [file mmc5.docx]

**Targeting the ERK signaling pathway to halt adventitia remodeling of ascending thoracic aortic aneurysm**

**SUPPLEMENTAL MATERIAL**

**Suppl. Table 3**. **Primary antibodies used for immunohistochemistry on human and mouse tissues.**

| **Target** | **Host** | **Dilution** | **Company & CAT#** |
| --- | --- | --- | --- |
| **PDGFRβ (anti-human)** | Goat | 1:50 | R&D AF385 |
| **PDGFRβ (anti-mouse)** | Goat | 1:50 | R&D AF1042 |
| **Active MMP9** | Mouse | 1:200 | NovusBio NBP2-13173 |
| **αSMA** | Mouse (conjugated) | 1:400 | Dako M0851 |
| **CD31** | Rabbit | 1:20 | Abcam Ab28364 |
| **CD45** | Rat | 1:500 | NovusBio NB100-77417 |
