# Supplementary material for: Targeting the ERK Signaling Pathway to Halt Adventitia Remodeling of Ascending Thoracic Aortic Aneurysm
Source: JACC Basic Transl Sci. 2026 Sep 25;11(10):101665. doi: 10.1016/j.jacbts.2026.101665 (PMC13635404; doi:10.1016/j.jacbts.2026.101665)
Supplement: Supplementary file 6 [file mmc6.docx]

**Targeting the ERK signaling pathway to halt adventitia remodeling of ascending thoracic aortic aneurysm**

**SUPPLEMENTAL MATERIAL**

**Suppl. Table 4**. **Primary antibodies used for immunocytochemistry in human cells.**

| **Primary Ab** | **Host** | **Company & CAT#** | **Dilution** | **Triton-X100**  **(permeabilization)** |
| --- | --- | --- | --- | --- |
| **CD31** | Rabbit | Abcam Ab28364 | 1:50 | No |
| **CD146** | Rabbit | Abcam Ab75769 | 1:100 | Yes |
| **NG2** | Rabbit | Millipore AB5320 | 1:50 | No |
| **PDGFRβ** | Goat | R&D AF385 | 1:50 | No |
| **PDGFRα** | Mouse | Santa Cruze sc-398206 | 1:100 | Yes |
| **SM α-Calponin** | Rabbit | Abcam Ab46794 | 1:100 | Yes |
| **α-SM-MHC11** | Rabbit | Abcam Ab53219 | 1:50 | Yes |
| **Smoothelin B** | Rabbit | Santa Cruz sc-28562 | 1:100 | Yes |
| **α-SMA** | Mouse | Dako M0851 | 1:100 | Yes |
| **Transgelin (SM22α)** | Mouse | Santa Cruz sc-271719 | 1:50 | Yes |
| **Vimentin** | Rabbit | Abcam Ab92547 | 1:500 | Yes |
| **Ki67** | Rabbit | Abcam, ab16667 | 1:500 | Yes |
| **P-ERK (Thr202/ Tyr204)** | Rabbit | Cell Signalling, #4370 | 1:200 | Yes (Methanol) |
