# Supplementary material for: Targeting the ERK Signaling Pathway to Halt Adventitia Remodeling of Ascending Thoracic Aortic Aneurysm
Source: JACC Basic Transl Sci. 2026 Sep 25;11(10):101665. doi: 10.1016/j.jacbts.2026.101665 (PMC13635404; doi:10.1016/j.jacbts.2026.101665)
Supplement: Supplementary file 7 [file mmc7.docx]

**Targeting the ERK signaling pathway to halt adventitia remodeling of ascending thoracic aortic aneurysm**

**SUPPLEMENTAL MATERIAL**

**Suppl. Table 5**. **List of RT-qPCR probes used in in vitro cell studies.**

| **Tested Gene** | **ID** |
| --- | --- |
| ***MMP 2*** | HS01548727 |
| ***MMP 9*** | HS00234579 |
| ***TIMP 2*** | HS00234278 |
| ***GAPDH* (Housekeeping)** | HS02786624 |
