# Supplementary material for: Targeting the ERK Signaling Pathway to Halt Adventitia Remodeling of Ascending Thoracic Aortic Aneurysm
Source: JACC Basic Transl Sci. 2026 Sep 25;11(10):101665. doi: 10.1016/j.jacbts.2026.101665 (PMC13635404; doi:10.1016/j.jacbts.2026.101665)
Supplement: Supplementary file 8 [file mmc8.docx]

**Targeting the ERK signaling pathway to halt adventitia remodeling of ascending thoracic aortic aneurysm**

**SUPPLEMENTAL MATERIAL**

**Suppl. Table 6**. **List of antibodies used for Western Blot.**

| **Target** | **Host** | **Dilution** | **Company & CAT#** |
| --- | --- | --- | --- |
| **ERK 1/2 (p44/42 MAP)** | Rabbit | 1:1000 | Cell Signalling, #4695 |
| **P-ERK (Thr202/ Tyr204)** | Rabbit | 1:2000 | Cell Signalling, #4370 |
| **β-Tubulin** | Mouse | 1:1000 | Cell Signalling, #86298 |
